# Metabolomic Insights into Lysosomal Storage Diseases: An Untargeted View

**DOI:** 10.3390/metabo16070480

**Published:** 2026-07-08

**Authors:** Gessica Di Carlo, Maria Lucia Tommolini, Alberto Frisco, Giorgia Spalluto, Dominic Foley, Mirco Zucchelli, Maria Concetta Cufaro, Ilaria Cicalini, Ines Bucci, Luca Federici, Vincenzo De Laurenzi, Damiana Pieragostino, Claudia Rossi

**Affiliations:** 1 Center for Advanced Studies and Technology (CAST), “G. d’Annunzio” University of Chieti-Pescara, 66100 Chieti, Italy; gessica.dicarlo@phd.unich.it (G.D.C.); maria.tommolini@unich.it (M.L.T.); alberto.frisco@unidav.it (A.F.); giorgia.spalluto@phd.unich.it (G.S.); m.zucchelli@unich.it (M.Z.); maria.cufaro@unich.it (M.C.C.); ilaria.cicalini@unich.it (I.C.); ines.bucci@unich.it (I.B.); luca.federici@unich.it (L.F.); vincenzo.delaurenzi@unich.it (V.D.L.); damiana.pieragostino@unich.it (D.P.); 2 Department of Science, “G. d’Annunzio” University of Chieti-Pescara, 66100 Chieti, Italy; dominic_foley@waters.com; 3 Department of Innovative Technologies in Medicine and Dentistry, “G. d’Annunzio” University of Chieti-Pescara, 66100 Chieti, Italy; 4 Department of Human, Legal and Economic Sciences, Telematic University of “Leonardo Da Vinci”, 66010 Torrevecchia Teatina, Italy; 5 Department of Neuroscience, Imaging and Clinical Sciences, “G. d’Annunzio” University of Chieti-Pescara, 66100 Chieti, Italy; 6 Department of Medicine and Aging Science, “G. d’Annunzio” University of Chieti-Pescara, 66100 Chieti, Italy

**Keywords:** lysosomal storage diseases, untargeted metabolomics, sphingolipidoses, mucopolysaccharidoses, Pompe disease, oligosaccharidosis, mucolipidoses, lysosomal proteinoses

## Abstract

Lysosomal Storage Diseases (LSDs) include roughly 70 inherited metabolic disorders, most of which are expressed in an autosomal recessive pattern. These conditions arise from mutations in genes encoding lysosomal enzymes, leading to intracellular buildup of substrates and subsequent lysosomal dysfunction. LSDs can be broadly classified based on the nature of the stored substrate, encompassing Sphingolipidoses, Mucopolysaccharidoses, Lysosomal Glycogen Storage Disease, Oligosaccharidoses, Mucolipidoses, and Lysosomal Proteinoses. Although individually rare, LSDs collectively affect approximately 1 in 5000 live births. They usually manifest in childhood, but adult-onset types are also detected. Clinical manifestations are heterogeneous and may involve the central nervous system, skeletal system, skin, heart, muscles, kidneys, and other organs. Several therapeutic strategies are available for LSDs, including Enzyme Replacement Therapy to restore deficient enzymes, Hematopoietic Stem Cell Transplantation to provide functional donor-derived cells, Substrate Reduction Therapy and pharmacological chaperones to modulate substrate turnover or enhance enzyme stability, alongside symptomatic and supportive treatments. Ongoing research is also exploring gene therapy-based strategies. Current diagnostic criteria remain insufficient for reliable presymptomatic diagnosis, highlighting the need for more sensitive and specific approaches. In this context, untargeted metabolomics is a powerful strategy to investigate pathogenic pathways, identify novel diagnostic and prognostic biomarkers, and uncover potential therapeutic targets. Accordingly, this review provides an overview of LSDs, focusing on untargeted metabolomics studies and their contribution to the discovery of novel biomarkers and previously unrecognised pathogenic mechanisms relevant to diagnostic and therapeutic innovation.

## 1. Introduction

Lysosomal Storage Diseases (LSDs) are a heterogeneous group of inherited metabolic disorders caused by defects in lysosomal function, leading to the progressive accumulation of undegraded substrates within cells and tissues [1]. As part of the broader class of inborn errors of metabolism (IEMs), LSDs are characterised by complex and multisystemic clinical manifestations, frequently involving progressive neurological impairment, inflammation, mitochondrial dysfunction, and altered lipid metabolism [1,2]. Despite advances in molecular and enzymatic diagnostics, the clinical heterogeneity and overlapping phenotypes of LSDs continue to make early diagnosis and disease monitoring challenging.

In recent years, untargeted metabolomics, one of the most recent members of the “omics” family, has emerged as a promising approach for the comprehensive characterisation of metabolic alterations associated with IEMs and LSDs. Metabolomic changes are truly impressive in this underappreciated field, so untargeted metabolomics, unlike targeted approaches currently used in newborn screening and routine diagnostics, enables the simultaneous analysis of a broad range of metabolites without predefined targets, thereby providing a more global overview of dysregulated biochemical pathways [2]. Over the past few decades, significant progress has been made in the identification and quantification of metabolites in biological fluids and samples, especially due to technological advances in mass spectrometry (MS) and computational models in bioinformatics tools. In general, the powerful ability of metabolomics to measure low-molecular-weight metabolites associated with a disease phenotype may be exploited to unravel dynamic changes in living organisms, potentially enabling us to understand metabolic pathways involved in LSDs for monitoring disease progression and therapies. As a matter of fact, this strategy may improve biomarker discovery, patient stratification, and therapeutic monitoring, while also contributing to a deeper understanding of LSDs pathophysiology by unravelling the biological mechanisms behind these metabolic shifts.

**Figure 1:**
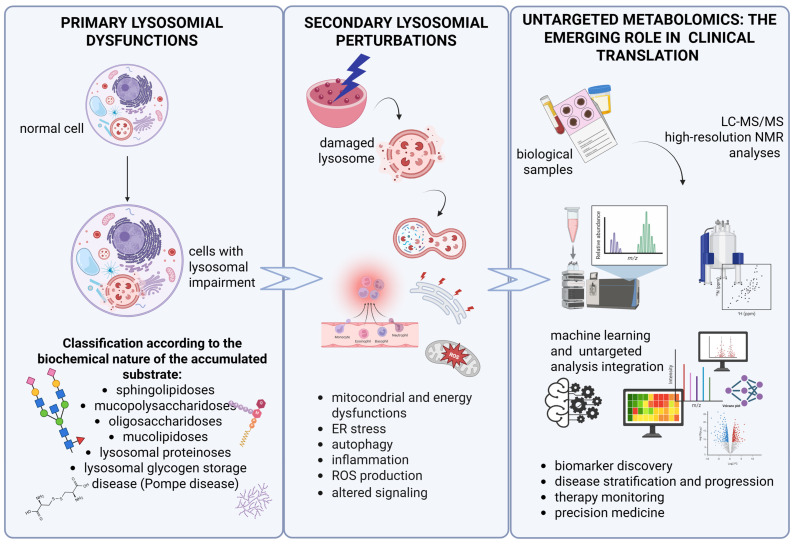
Schematic overview of the review objective and scope. Figure is divided into three sections, highlighting the cross-talk between substrate accumulation across different classes of LSDs and the secondary perturbations occurring at the organismal level. Untargeted metabolomics represents the connecting framework, providing insights into disease mechanisms and addressing key questions related to the identification and translation of novel putative biomarkers. Created in https://BioRender.com.

In this review, we summarised the current applications of untargeted metabolomics in LSDs, highlighting common metabolic signatures and their potential translational relevance. Furthermore, the clinical manifestations of LSDs are discussed according to the nature of the accumulated substrate, including sphingolipidoses, mucopolysaccharidoses, oligosaccharidoses, mucolipidoses, lysosomal proteinoses and lysosomal glycogen storage disease. On the one hand we aimed to explore the clinical utility and the translational perspective of untargeted metabolomics approach for each class of LSDs provided below. On the other hand, we described how the combination between MS platforms and machine learning algorithms has recently contributed to build new data-driven experimental workflows for LSDs research with a view to translating novel putative biomarkers into larger panels in multiplex analytical assays. The bibliographic search strategy was based on the following search terms. We selected both original scientific articles (including studies on cellular models, murine models and clinical cases) and reviews on PubMed/MEDLINE website with primary focus on studies published within the last decade. Studies were selected based on their relevance to metabolomic biomarkers in LSDs, whereas editorials, conference abstracts, duplicate records, and articles not directly related to the topic were excluded, for example study on LSDs in which a proteomics approach was performed. Then, we obtained about 382 eligible paper using as searching keywords “lysosomal storage disorders”, “metabolomics”, “metabolites” (combined with each other using the Boolean operator -AND -OR), We then narrowed our literature search to the specific LSD subclasses by including the names of individual LSDs and “untargeted metabolomics” as an additional keyword, identifying 129 relevant articles that were included in the final version of the bibliography. At the end, we selected the articles according to emerging role of untargeted metabolomics in clinical translation. In this regard, Figure 1 summarises the main objectives of this review. For each class of LSDs, we will first discuss the pathological mechanisms and the secondary lysosomal perturbations triggered by substrate accumulation. We will then explore the emerging role of untargeted metabolomics as a powerful and innovative strategy to reveal hidden metabolic signatures and identify novel mechanisms underlying secondary lysosomal dysfunction, thereby expanding our understanding of disease biology and opening new avenues for translational applications.

## 2. Sphingolipidoses

Sphingolipidoses are a subgroup of LSDs caused by deficiencies or dysfunctions of enzymes involved in sphingolipid degradation. Sphingolipids are defined as sphingosine-based membrane lipids that play essential roles in cellular structure, homeostasis, signalling, development, and cell fate regulation. In these disorders, impaired catabolism leads to the progressive intracellular accumulation of sphingolipids, reaching toxic levels that disrupt cellular function and result in multisystemic pathology [3,4].

### 2.1. Krabbe Disease

Krabbe Disease (KD), also known as Globoid Cell Leukodystrophy, is a rare autosomal recessive Lysosomal Storage Disease (LSD) characterised by progressive neurodegeneration resulting from a deficiency of the lysosomal enzyme galactocerebrosidase (GALC), which plays a key role in myelin turnover. Pathogenic variants in the *GALC* gene reduce enzymatic activity, leading to the accumulation of the cytotoxic sphingolipid psychosine (PSY), a major contributor to widespread demyelination, cerebral white matter degeneration, and peripheral neuropathy. The estimated incidence of KD ranges from 0.3 to 2.6 cases per 100,000 live births [5]. KD is commonly classified according to the age of onset into early-infantile (EIKD; <6 months), late-infantile (LIKD; 6–12 months), juvenile (before 15 years of age) and adult-onset (after mid-adolescence) forms. Early manifestations include irritability, unexplained fever, limb stiffness, seizures, feeding difficulties, gastroesophageal reflux, and progressive psychomotor delay. Disease progression is associated with severe neurological impairment, including muscle weakness, spasticity, hearing loss, and visual deterioration [6]. Currently, presymptomatic hematopoietic stem and progenitor cell transplantation (HSPCT) represents the only available therapeutic strategy capable of mitigating central nervous system pathology in KD patients [7]. Several investigational approaches are under preclinical evaluation, including gene therapy (GT), chaperone-mediated therapy, and nanocarrier-based enzyme replacement therapy (ERT) [8]. The diagnostic workflow for KD relies on a sequential strategy that begins with screening for GALC enzymatic activity [9], followed by PSY quantification as a second-tier confirmatory test [10].

Advanced omics approaches have increasingly contributed to the characterisation of metabolic and lipid alterations associated with KD pathogenesis.

LC–MS-based comprehensive lipidomic profiling of nervous system tissues, including sciatic nerve (SN), spinal cord, brain, and cerebellum, in homozygous Twitcher mice (HOM), a widely used model of KD, identified profound lipid remodelling compared with wild-type mice [8]. HOM mice showed an overall reduction in lipid abundance, particularly in SN, consistent with the severe demyelinating and neurodegenerative phenotype of the disease. Myelin-associated lipid classes, including hexosylceramides (HexCer), sulfatides (SHexCer), phosphatidylcholines (PC), and ether-linked phosphatidylethanolamines (PE-O), were markedly decreased, suggesting major alterations in myelin composition and membrane integrity. Interestingly, the reduction in HexCer and SHexCer was described as an unexpected finding despite their abundance in myelin. Although this observation may reflect the extensive demyelination and lipid remodelling characteristic of KD, methodological factors related to lipid normalisation and annotation were also acknowledged as potential contributors to the observed decrease. PC depletion may additionally reflect oxidative conversion into neurotoxic oxidised phosphatidylcholines (OxPC). Significant dysregulation of diacylglycerol (DG) levels was also observed, potentially contributing to altered membrane biophysical properties due to its role as both a metabolic intermediate and secondary lipid messenger.

Consistent with the extensive lipid remodelling observed in HOM tissues, PSY levels were markedly elevated and closely interconnected with ceramide (Cer) and HexCer metabolism, supporting the impact of GALC deficiency on sphingolipid homeostasis. PSY accumulation has also been associated with disruption of sphingomyelin-enriched membrane microdomains, increased membrane rigidity, and enhanced microvescicle shedding, all of which may contribute to myelin instability. Correlation analyses further demonstrated predominantly negative associations between PSY and multiple lipid classes, including phospholipids, sphingomyelin, Cer, HexCer, SHexCer, and DG, whereas positive correlations were observed for cholesteryl esters and phosphatidylglycerol.

Complementary metabolomic analyses further support these findings. Rhombencephalic regions from Twitcher and wild-type (WT) mice were profiled using LC–MS and GC–MS at two timepoints (P15 and P22), corresponding to pre- and post-symptomatic stages of the disease. A total of 314 metabolites were identified per sample, with substantial variation between P15 and P22 reflecting active brain maturation and myelination [11].

Between-group comparisons revealed limited differences at the pre-symptomatic stage, which became markedly more pronounced following disease onset. In this context, alterations in lipid and membrane metabolism emerged, including reduced glycerol-3-phosphate and glycerophosphocholine levels alongside increased CDP-choline at P15, suggestive of early compensatory responses. Conversely, decreased cholesterol precursors and N-acetylaspartate (NAA) at P22 were consistent with reduced myelin turnover. Overall, these findings indicate an early disruption of membrane homeostasis involving glycerophospholipids, sphingolipids, cholesterol, and myelin.

Analyses conducted by Nadav I. Weinstock et al. further revealed extensive metabolic alterations associated with KD progression [11]. At the pre-symptomatic stage (P15), glucose hypometabolism was evident, with reduced glycolytic and pentose phosphate pathway intermediates together with decreased mannose-6-phosphate, suggesting impaired lysosomal transport. At the post-symptomatic stage (P22), increased 1,5-anhydroglucitol further supported disrupted glucose homeostasis.

Marked alterations in energy and mitochondrial metabolism also emerged at P22, including elevated branched-chain amino acids and increased BCAA-derived acylcarnitines (acetylcarnitine, propionylcarnitine, isobutyrylcarnitine, 2-methylbutyrylcarnitine [C5], β-hydroxyisovalerylcarnitine, butyrylcarnitine, hydroxybutyrylcarnitine, and 3-dehydrocarnitine), indicating impaired mitochondrial metabolism and incomplete BCAA oxidation. Perturbations in carnitine metabolism further supported this energetic impairment.

In parallel, signatures of neuroinflammation and oxidative stress emerged, including increased corticosterone, 12-HETE, and α-tocopherol levels, together with markers of extracellular matrix remodelling. Dysregulation of several neurotransmitters and their precursors, including tryptophan, phenylalanine, putrescine, and serine, further indicated altered neurotransmission.

### 2.2. Gaucher Disease

Gaucher Disease (GD) is an autosomal recessive genetic disorder with an estimated incidence ranging from 0.4 to 5.8 per 100,000 individuals. It is caused by a deficiency of the lysosomal enzyme glucocerebrosidase (GBA), which catalyses the hydrolysis of the glycolipid glucocerebroside into Cer and glucose. This enzymatic defect leads to the accumulation of its substrate, glucosylceramide, within lysosomal macrophages, resulting in multisystemic manifestations involving the liver, spleen, bone marrow, lungs, and central nervous system, and clinically presenting with splenomegaly, haematological abnormalities, and skeletal disorders [12,13]. GD is traditionally classified into three clinical subtypes: type I (non-neuronopathic form, primarily characterised by visceral involvement), type II (acute neuronopathic form with early-onset neurodegeneration), and type III (chronic neuronopathic form with later-onset neurological symptoms) [14,15]. Therapeutic approaches for GD include ERT and substrate reduction therapy, which effectively improves visceral symptoms but has limited impact on neurological involvement [12]. Newborn screening for GD relies on first-tier measurement of GBA activity in dried blood spots (DBS), followed by second-tier analysis of glucosylsphingosine (Lyso-Gb1), which improves diagnostic specificity [16,17,18].

Untargeted metabolomic approaches have provided valuable insights into the biochemical complexity of GD beyond the accumulation of glucosylceramide and LysoGb1. Using high-resolution plasma metabolomics, Menkovic et al. identified a disease-specific metabolic signature characterised not only by elevated LysoGb1 and several LysoGb1 analogues, but also by significant alterations in other sphingolipid-related metabolites, including sphingosylphosphorylcholine and N-palmitoyl-O-phosphocholineserine [19]. These findings indicate that GBA deficiency affects multiple interconnected metabolic pathways, extending beyond the primary lysosomal storage defect. Importantly, the identified metabolomic profile enabled separation of GD patients and healthy controls within multivariate statistical models, supporting the potential of untargeted metabolomics as a complementary tool for disease characterisation, biomarker discovery, and patient monitoring.

In this context, although alterations in acylcarnitine species have occasionally been reported in untargeted metabolomic studies, these findings have not been consistently replicated and no plasma acylcarnitine has emerged as a validated biomarker for Gaucher disease.

Multisystem alterations associated with GD have been further investigated using zebrafish models generated through TALEN-mediated *GBA1* loss of function, providing early insights into the systemic consequences of GBA deficiency. These models recapitulate both visceral and neural phenotypes observed in human disease, representing one of the first vertebrate systems to do so comprehensively [15].

At the neurological level, disease progression is associated with early microglial activation and increased expression of the inflammatory microRNA miR-155, suggesting the presence of a conserved neuroinflammatory signature.

Beyond the nervous system, disruption of *GBA1* has also been linked to impaired osteoblast differentiation and downregulation of canonical Wnt signalling, indicating a potential role of lysosomal dysfunction in skeletal abnormalities.

### 2.3. Fabry Disease

Fabry Disease (FD) is a rare X-linked LSD caused by deficiency of the enzyme α-galactosidase A (GLA), whose reduced or absent activity leads to progressive accumulation of glycosphingolipids with terminal α-D-galactosyl residues. In particular, globotriaosylceramide (Gb3) accumulates within lysosomes and is partially converted into (Lyso-Gb3) by acid ceramidase; both metabolites can be detected in plasma, urine, and multiple tissues, including cardiac, renal, endothelial, and nervous systems [20,21,22]. The estimated incidence ranges from 1 in 40,000 to 1 in 117,000 individuals [23]. Clinically, FD is characterised by a multisystem involvement affecting the heart, kidneys, vasculature, skin, and gastrointestinal tract. Neurological manifestations include acroparesthesias, pain crises, autonomic dysfunction with hypohidrosis, gastrointestinal dysmotility, and cerebrovascular complications such as transient ischemic attacks, stroke, and white matter lesions related to small vessel disease. Renal impairment represents a major determinant of disease severity [24,25]. Two main phenotypes are recognised: a classic early-onset form and a later-onset variant with predominant single-organ involvement [26]. ERT based on recombinant agalsidase has demonstrated beneficial effects on clinical outcomes, biomarkers, and disease progression, improving quality of life in FD patients [23,26]. Diagnosis relies on assessment of GLA enzymatic activity and genetic analysis of the *GLA* gene. In males, detection of reduced enzyme activity in plasma, leukocytes, or DBS typically enables a diagnosis, followed by molecular confirmation. In females, due to X-chromosome inactivation, enzymatic activity may be normal, making genetic testing the gold standard. Lyso-Gb3 represents a key biomarker for diagnosis and therapeutic monitoring, particularly in late-onset variants with residual enzyme activity [22,26].

Urinary Gb3 is a traditional biomarker used to monitor treatment response in FD, as its levels decrease following ERT, although it is not considered a reliable surrogate endpoint in clinical studies. Gb3 reduction can be detected as early as two weeks after ERT initiation, while anti–α-galactosidase A antibodies have been associated with increased urinary Gb3 levels, reflecting treatment-related immunogenicity. Conventional urinary biomarkers, including proteinuria and albuminuria, are widely used indicators of glomerular and tubular dysfunction and strong predictors of renal involvement and disease progression, although renal damage may already be present in the non-albuminuric stage. Additional emerging markers of renal injury, such as uromodulin, N-acetyl-β-D-glucosaminidase, and β2-microglobulin, are currently under investigation.

Metabolomic studies have significantly expanded the urinary biomarker landscape in FD, identifying novel metabolites including seven Lyso-Gb3 analogues with modified sphingosine moieties [26]. While Lyso-Gb3 is mainly elevated in plasma, its analogues are more abundant in urine, and the combined urinary levels of Lyso-Gb3 and derivatives show high specificity for FD. Similarly, increased urinary galabiosylceramide (Ga2) isoforms have been reported in FD patients, with six Ga2-related species detected exclusively in affected individuals, supporting their diagnostic potential [22]. Comprehensive LC–MS/MS glycosphingolipidomic profiling has further revealed a broader metabolic remodelling, with long-chain ceramide dihexoside isoforms significantly elevated in urine and showing superior ability to discriminate female patients, including asymptomatic carriers, compared with Gb3 and Lyso-Gb3 [23].

These urinary findings are complemented by plasma metabolomic profiling. Evidence from multiple studies suggests that quantification of Lyso-Gb3 analogues provides greater insight into FD status and treatment response than Gb3 or Lyso-Gb3 alone. LC–TOF-MS/MS plasma metabolomic profiling has identified novel Gb3-related biomarkers, including sphingosine- and fatty acid–isomeric forms and methylated Gb3 derivatives, which effectively discriminate FD patients from controls. These methylated species are proposed to represent intermediates in the deacylation pathway leading to Lyso-Gb3, a hypothesis supported by analogous observations in Ga2 metabolism. More recently, network-based targeted metabolomic approaches have identified a 13-metabolite plasma signature, including markers of oxidative stress and glycerophospholipid metabolism, reinforcing the central role of lipid remodelling and redox imbalance in FD [23].

Beyond metabolomic alterations, circulating biomarkers underscore the multisystem complexity of FD, involving inflammation, cardiac remodelling, and metabolic dysregulation. Pro-inflammatory mediators, including tumour necrosis factor (TNF), interleukin-6 (IL-6), and TNF receptors 1 and 2 (TNFR1/2), are significantly elevated in FD patients, supporting the role of chronic inflammation in disease pathophysiology. Cardiac biomarkers such as BNP and MR-proANP are increased in patients with late gadolinium enhancement and diastolic dysfunction, reflecting progressive cardiac remodelling. Consistently, matrix metalloproteinases MMP2 and MMP9 are associated with structural remodelling, with MMP2 linked to heart failure with preserved ejection fraction (HFpEF) and MMP9 to left ventricular hypertrophy and extracellular matrix disruption. Among circulating biomarkers, Lyso-Gb3, NT-proBNP, and MMP9 are consistently elevated, reflecting both substrate accumulation and fibrotic processes. At the molecular level, pathway analyses have revealed upregulation of glycolysis, lysosomal function, and sphingolipid metabolism in FD patients compared with controls, together with increased β-galactosidase expression in treatment-naïve individuals, suggesting a role in glycosphingolipid accumulation [26].

### 2.4. Metachromatic Leukodystrophy

Metachromatic Leukodystrophy (MLD) is a rare LSD caused by biallelic pathogenic variants in the *ARSA* gene encoding arylsulfatase A (ASA), leading to deficient or absent enzymatic activity. This results in lysosomal accumulation of sulfatides (3-O-sulfogalactosylceramides), which predominantly accumulate in the central and peripheral nervous systems, particularly in oligodendrocytes and myelin, causing progressive demyelination and neurodegeneration. Although MLD is a multisystem disorder that may involve the kidneys, liver, and gallbladder, neurological impairment represents the dominant clinical feature. The disease is classified by age of onset into late-infantile (<30 months), early-juvenile (2.5–6 years), late-juvenile (7–16 years), and adult (>16 years) forms, with an incidence of 0.16–1.85 per 100,000 live births [27,28]. The late-infantile form is rapidly progressive, with loss of motor function within approximately 40 months and death typically within 5 years, whereas juvenile and adult forms show a slower but continuously progressive course with worsening after loss of ambulation. Early stages, particularly in later-onset forms, are characterised by behavioural and psychiatric symptoms. Clinically, motor-, cognitive-, and mixed phenotypes are recognised, including motor delay, gait abnormalities, pyramidal signs, and cognitive-behavioural disturbances such as irritability, learning difficulties, and mood changes [27]. MLD diagnosis relies on clinical evaluation, neuroimaging, biochemical testing, and genetic confirmation. Characteristic MRI findings are supported by reduced ASA activity in leukocytes or fibroblasts, increased urinary sulfatides, and *ARSA* gene analysis. Newborn screening follows a three-tier DBS-based strategy, including LC–MS/MS quantification of C16:0 sulfatides, followed by ASA enzymatic testing and confirmatory genetic analysis. At the pathophysiological level, sulfatide accumulation induces lysosomal dysfunction, membrane damage, and release of intracellular contents, leading to oligodendrocyte death, demyelination, and inflammatory activation mediated by cytokine and nitric oxide–related pathways [27]. Therapeutically, allogeneic hematopoietic stem cell transplantation (HSCT) remains the standard of care for presymptomatic or early-stage juvenile and adult patients, although efficacy is limited once neurological symptoms are established. Emerging approaches include ex vivo lentiviral hematopoietic stem cell gene therapy (atidarsagene autotemcel) for presymptomatic and early symptomatic patients, and intrathecal recombinant human arylsulfatase A (rhASA), aimed at improving CNS enzyme delivery compared with intravenous ERT [27,28].

In this context, the MLD mouse model (*ARSA*^−^/^−^), characterised by loss of ASA activity and progressive sulfatide accumulation, represents a valuable system for investigating disease-associated lipid remodelling. Untargeted lipidomic analysis of brain-derived extracellular vesicles (EVs) across disease progression (P30, 3 and 6 months) revealed progressive and widespread lipid remodelling, with an increasing number of altered species over time, reflecting worsening metabolic dysregulation.

A marked accumulation of SHexCer, particularly short-chain species (≤18 carbon atoms), was observed and, for the first time, confirmed within brain-derived EVs. In parallel, ceramides were significantly dysregulated, with decreased non-hydroxylated (Cer_NS) and dihydroceramides (Cer_NDS) and increased α-hydroxylated species (Cer_AS), indicating an imbalance in sphingolipid metabolism. Consistently, increased HexCer and their dihydro derivatives further supported broader sphingolipid remodelling. Beyond sphingolipids, extensive alterations affected multiple lipid classes, including decreased fatty acids, FAHFAs, and acylglycerols (DG and TG), suggesting impaired energy metabolism and lipid turnover. In contrast, brain-derived extracellular vesicles from MLD mice showed increased lysophosphatidylcholines (LPCs) and increased acylcarnitine isoforms consistent with mitochondrial dysfunction and/or fatty acid overload, with LPCs potentially contributing to demyelination processes. Additional changes in glycerophospholipids, including reduced PE, PS, PI, and PC levels, together with alterations in PG and BMP, were consistent with findings previously reported in human MLD patients. Overall, these results highlight extensive EV-associated lipid remodelling during MLD progression and identify fatty acids, FAHFAs, and acylglycerols as potential biomarkers of disease progression [29].

Additional urinary metabolomic studies using proton nuclear magnetic resonance spectroscopy (^1^H-NMR) have further supported these findings, revealing extensive metabolic remodelling in MLD patients [28]. Increased levels of NAA, a marker of neurodegeneration, were observed, together with distinct metabolic signatures between early- and late-onset forms, characterised by a shift toward ketogenesis and reduced tricarboxylic acid (TCA) cycle intermediates. Paediatric patients showed elevated ketone bodies and markers of impaired energy metabolism, whereas 3-hydroxy-3-methylglutarate (3-HMG) was consistently reduced across all subtypes [28]. Complementary magnetic resonance spectroscopy studies in juvenile and adult-onset patients further supported the prognostic relevance of NAA, showing strong associations between baseline NAA levels and motor function at follow-up. Reduced NAA and glutamate, together with increased lactate, myo-inositol, and choline-containing compounds, were associated with worse clinical outcomes and active demyelination [27]. Urinary metabolomic profiling also revealed increased markers of intestinal and/or urinary dysbiosis, including trimethylamine (TMA), L-citramalic acid, and 2-furoylglycine, suggesting microbiota-related metabolic involvement. Subgroup analyses further showed elevated methylguanidine and lactic acid in late-onset forms, whereas juvenile patients achieving clinical stabilisation after HSCT exhibited increased neopterin, consistent with macrophage activation and potential disease stabilisation [28]. Overall, these findings identify NAA as a consistent biomarker of neurodegeneration across biofluids and imaging modalities and indicate that urinary metabolic profiles reflect both central nervous system involvement and systemic as well as microbiota-related alterations, with potential prognostic value for disease progression and therapeutic response [28].

Further refinements in biochemical screening have been proposed. The inclusion of the short-chain sulfatide 16:1-OH has recently emerged as a complementary biomarker for first-tier biochemical screening of MLD. Combined quantification of 16:1-OH and the conventional 16:0-OH resulted in an approximately 20-fold reduction in false-positive cases requiring second-tier testing, without affecting sensitivity. These results indicate that simultaneous measurement of both sulfatide species improves the accuracy and specificity of initial screening workflows, enhancing diagnostic efficiency [27,30,31].

### 2.5. GM1-Gangliosidoses

GM1 Gangliosidosis is an autosomal recessive LSD caused by mutations in the *GLB1* gene, which encodes β-galactosidase, leading to the progressive accumulation of GM1 ganglioside and related substrates. The disease primarily affects the central nervous system but may also involve peripheral tissues, resulting in a multisystem neurodegenerative disorder. It shows a broad clinical spectrum and is classified into infantile, juvenile, and adult forms (types 1–3), reflecting age at onset and severity. The infantile form is rapidly progressive, whereas the juvenile form is characterised by slower but progressive neurological deterioration with variable systemic involvement. The estimated incidence is approximately 1 in 100,000–200,000 live births. Currently, no curative therapies are available, and management remains supportive. Diagnosis relies on clinical evaluation, enzymatic assays, and genetic testing, although early recognition is often limited by nonspecific symptoms and disease rarity. These limitations have driven increasing interest in metabolomic biomarkers to improve early detection, disease stratification, and longitudinal monitoring, as well as to support emerging therapeutic strategies [32].

Proton (^1^H) NMR-based metabolomics of biofluids, including plasma, cerebrospinal fluid (CSF), and urine, combined with multivariate statistical approaches, represents a robust platform for investigating systemic metabolic alterations and identifying disease-associated biomarkers. In a recent study, plasma ^1^H-NMR metabolomics was applied to patients with GM1 gangliosidosis type 2 (GM1T2) compared with healthy controls, revealing a distinct disease-associated metabolic profile indicative of systemic metabolic reprogramming. Multivariate and metabolite set enrichment analyses performed in GM1T2 patients highlighted increased levels of amino acids, including branched-chain (valine, leucine, isoleucine) and aromatic amino acids (tyrosine, phenylalanine), together with glutamine and histidine, suggesting altered amino acid and nitrogen metabolism. Elevated lactate, formate, and urea indicated a shift toward anaerobic energy metabolism, while changes in creatine, phosphocreatine, and creatinine reflected impaired energy buffering and homeostasis. In contrast, reduced triglycerides suggested a hypolipidemic plasma profile. Pathway analysis confirmed alterations in lipid metabolism, fatty acid biosynthesis, and mitochondrial function, including β-oxidation and the TCA cycle, consistent with mitochondrial dysfunction, oxidative stress, and impaired antioxidant defences. Overall, lactate, branched-chain amino acids, and creatine-related metabolites emerged as the most consistent discriminant features [32].

### 2.6. GM2-Gangliosidoses

GM2 Gangliosidoses, including Tay–Sachs and Sandhoff disease (SD), are inherited LSDs of glycosphingolipid catabolism characterised by the accumulation of sialic acid–containing gangliosides in the central nervous system, leading to progressive neurodegeneration. They result from defects in the β-hexosaminidase system: Tay–Sachs disease is caused by mutations in *HEXA*, SD by mutations in *HEXB*, leading to combined β-hexosaminidase A and B deficiency, while a rare AB variant is associated with mutations in *GM2A*, encoding the GM2 activator protein. Clinically, GM2 gangliosidoses show a broad spectrum of severity. Infantile-onset forms are characterised by early neurodevelopmental delay, hypotonia, dysphagia, and seizures, with rapid progression and early childhood mortality, whereas juvenile-onset forms follow a slower but progressive course, including ataxia, impaired coordination, dysarthria, hypotonia, and progressive neurological decline, ultimately leading to death in adolescence. No curative therapies are currently available, and management remains supportive, with an invariably progressive neurodegenerative course [33,34,35].

Neuroinflammatory processes further contribute to gangliosidosis pathogenesis. Serum and CSF samples from patients with gangliosidoses were analysed to investigate inflammation-related mediators associated with central nervous system involvement. Among 72 analytes, 13 were significantly elevated in CSF, whereas only osteopontin and insulin-like growth factor-binding protein 2 (IGFBP-2) were increased in serum, indicating a predominantly central inflammatory response. Notably, five inflammatory markers, epithelial-derived neutrophil-activating protein 78 (ENA-78), monocyte chemoattractant protein-1 (MCP-1), macrophage inflammatory protein-1α (MIP-1α), macrophage inflammatory protein-1β (MIP-1β), and tumour necrosis factor receptor 2 (TNFR2), were persistently elevated in the infantile phenotype, suggesting an association with more severe disease forms, a pattern not observed in attenuated phenotypes. These findings support a link between inflammatory activation and disease severity and highlight neuroinflammation as an active component in gangliosidosis pathogenesis. MCP-1 and MIP-1α have previously been associated with microglial activation in SD models, whereas ENA-78, MIP-1β, and TNFR2 may represent additional mediators of disease-associated inflammation. TNFR2 has been implicated in neuroprotective and remyelination processes, suggesting a potential compensatory response. Overall, the CSF inflammatory profile underscores the contribution of immune mediators to neurological injury and their potential utility as biomarkers for disease stratification and monitoring [33]. Complementary evidence from experimental models of SD further supports a role for neuroinflammation. Time-resolved analyses of central nervous system tissues revealed progressive glial activation, including increased myo-inositol levels and a reduced NAA/myo-inositol ratio, indicating a shift in neuronal-to-glial balance. Elevated lysophosphatidylcholine (LPC) species, known to promote microglial activation, demyelination, and inflammatory signalling, were also observed in brain and spinal cord, supporting a dynamic interplay between neuronal dysfunction and glial activation during disease progression [35].

In parallel with neuroinflammatory changes, SD exhibits extensive metabolic reprogramming across tissues. A global untargeted LC–MS metabolomic analysis of liver and brain tissues from a SD mouse model, together with human hippocampal samples, revealed extensive metabolic remodelling, with 177 dysregulated metabolites in mouse liver, 112 in brain, and 119 in human tissue. Brain and hippocampus were selected due to the prominent neurological involvement of the disease, whereas liver was included to capture systemic metabolic alterations in a central metabolic organ. Across tissues, a conserved metabolic signature emerged, characterised by alterations in amino acid and dipeptide metabolism consistent with increased protein catabolism, together with widespread lipid dysregulation. Reduced levels of polyunsaturated fatty acids, including docosahexaenoic acid and arachidonic acid, plasmalogen phosphatidylethanolamines, and HexCer were observed, together with altered lysophospholipid profiles and disruptions in sphingolipid and endolysosomal metabolism, including increased bis (monoacylglycero) phosphates (BMPs), suggestive of lysosomal dysfunction. Elevated sphingosine further indicated perturbation of sphingolipid salvage pathways. Pathway enrichment analyses highlighted alterations in glutamate and glutathione metabolism, reactive oxygen species (ROS) detoxification, and amino acid turnover, consistent with mitochondrial dysfunction and oxidative stress. Decreased NAA and neurotransmitters (glutamate, aspartate, and GABA), together with increased myo-inositol, reflected neuronal loss, impaired neurotransmission, and glial activation. Systemically, increased protein catabolism, redox imbalance, including elevated oxidised glutathione, and altered glycosaminoglycan metabolism, including N-acetylgalactosamine-4-sulfate (GalNAc4S), further supported widespread metabolic reprogramming. Collectively, these findings demonstrate that SD is characterised by interconnected metabolic disturbances across tissues, involving lipid homeostasis, neurotransmission, oxidative stress, and systemic metabolic adaptation [34,35].

Several metabolomic studies have identified candidate biomarkers reflecting both disease burden and pathogenic mechanisms in SD. Among lipid-related markers, the accumulation of GM2 and GA2 gangliosides, together with increased lysosomal-associated lipids such as BMPs, supports their use as indicators of impaired glycosphingolipid degradation and endolysosomal dysfunction. BMP (22:6/22:6) emerged as a sensitive marker of lysosomal stress and altered lipid trafficking, while elevated sphingosine levels indicated dysregulation of sphingolipid salvage pathways and altered neuronal signalling. Non-lipid metabolites further expanded the biomarker landscape. Reduced NAA, together with decreased glutamate, aspartate, and γ-aminobutyric acid (GABA), reflected neuronal loss, impaired neurotransmission, and mitochondrial dysfunction. Disturbances in NAA and glutamate metabolism, as well as altered aspartate availability, further suggested impaired mitochondrial–neuronal coupling and disrupted energy-dependent neurotransmitter cycling, supporting a generalised impairment of synaptic homeostasis associated with progressive neurocognitive decline. In parallel, increased oxidised glutathione and aminoadipic acid highlighted persistent oxidative stress and redox imbalance, whereas accumulation of GalNAc4S suggested impaired glycosaminoglycan turnover and broader lysosomal dysfunction. Collectively, these metabolites converge on interconnected pathological pathways, including mitochondrial dysfunction, neuroinflammation, defective lysosomal degradation, and oxidative stress responses. These findings support a multi-marker strategy integrating lipid, neurotransmitter, and redox-related metabolites for improved disease stratification, diagnosis, and therapeutic monitoring, providing a comprehensive framework linking metabolic alterations with disease progression [34,35].

### 2.7. Niemann-Pick Type C Disease

Niemann–Pick Disease type C (NPC) is a rare autosomal recessive LSD with an estimated incidence of approximately 1 in 100,000 individuals in Western populations. The disease is caused in ~95% of cases by mutations in *NPC1* and in ~5% by mutations in *NPC2*. NPC1 encodes a late endosomal/lysosomal transmembrane cholesterol transporter, whereas NPC2 encodes a soluble lysosomal cholesterol-binding protein involved in intracellular cholesterol transfer.

Defects in either protein impair intracellular lipid trafficking, leading to progressive accumulation of unesterified cholesterol and glycosphingolipids within late endosomes and lysosomes, the biochemical hallmark of the disease. Clinically, NPC is highly heterogeneous and typically characterised by progressive neurological decline, including ataxia, motor impairment, and cognitive deterioration, although adult-onset forms with predominant psychiatric manifestations are increasingly recognised. Despite the recent approval of symptomatic therapies such as arimoclomol and levacetylleucine, no curative or disease-modifying treatment is currently available.

At the molecular level, NPC is increasingly recognised as a disorder of endolysosomal lipid trafficking associated with profound sphingolipid dysregulation, supporting its role as a model disease for lysosomal lipid homeostasis dysfunction [36].

A hallmark of NPC is profound disruption of intracellular lipid trafficking leading to progressive lysosomal lipid storage. Targeted lipidomic studies in Npc1-deficient models have demonstrated marked accumulation of glycosphingolipids across multiple tissues, including GM2 and GM3 gangliosides, sphingoid bases, and monohexosylceramides, particularly in liver and brain, with levels correlating with disease progression severity. These findings support the central role of defective NPC1-mediated cholesterol egress in driving secondary sphingolipid storage and global endolysosomal lipid dysregulation. Endolysosomal dysfunction is further reflected by increased BMPs, lipid species enriched in late endosomal/lysosomal membranes, especially BMP (22:6/22:6), together with reductions in key myelin-associated lipids, including plasmalogen phosphatidylethanolamines and HexCer, indicating impaired membrane integrity and myelin maintenance from early disease stages. Elevated sphingosine species further suggest disruption of sphingolipid salvage pathways and altered protein kinase C (PKC)-dependent neuronal signalling relevant to synaptic stability. These findings identify NPC as a complex disorder of endolysosomal lipid homeostasis in which primary cholesterol trafficking defects trigger widespread disturbances in glycosphingolipid, sphingoid base, and membrane lipid metabolism. Importantly, pharmacological approaches such as 2-hydroxypropyl-β-cyclodextrin (HPβCD) and miglustat partially reversed several lipid abnormalities in both central and peripheral tissues, supporting the concept that lysosomal lipid storage in NPC represents a dynamic and potentially reversible metabolic process [37].

Beyond glycosphingolipid storage, NPC1 is characterised by broader sphingolipid dysregulation and accumulation of secondary bioactive lipids associated with lysosomal stress. Multi-tissue lipidomic studies identified consistent increases in alkyl-lysophosphatidylcholines (alkyl-LPCs), including LPC O-16:0, LPC O-18:1, and LPC O-18:0, across brain, liver, and other affected tissues in multiple NPC1 models, suggesting a conserved role in disease pathophysiology. Notably, alkyl-LPC accumulation occurred independently of classical platelet-activating factor (PAF) lipid alterations, supporting a selective dysregulation of lysophospholipid metabolism rather than generalised PAF pathway activation. These findings indicate that NPC1-associated lysosomal dysfunction extends beyond cholesterol and glycosphingolipid storage to involve broader remodelling of membrane-derived lipid mediators. Pharmacological treatment with HPβCD partially normalised alkyl-LPC levels in affected tissues, supporting their association with the underlying storage pathology and suggesting that these species reflect reversible components of lipid dyshomeostasis. In CSF, alkyl-LPCs showed dynamic treatment-responsive behaviour, with reduced levels in untreated patients and increased concentrations following HPβCD administration, particularly in clinically responsive individuals. Together, these investigations expand the NPC1 lipidome beyond classical storage lipids and identify alkyl-LPC species as potential biomarkers of lysosomal dysfunction, disease activity, and therapeutic response [38].

Alongside tissue lipid remodelling, several circulating biomarkers have emerged in NPC. The identification of reliable biofluid biomarkers remains a major challenge in NPC due to its clinical heterogeneity and the need for early minimally invasive diagnostic tools. Metabolomic and lipidomic studies have identified several circulating metabolites with diagnostic and translational potential in plasma, CSF and DBS. Targeted lipid profiling revealed consistent alterations in sphingolipid species, including increased plasma monoglycosylceramides and ceramides together with reduced sphingoid bases. Importantly, these lipid abnormalities respond to therapeutic interventions such as miglustat and HPβCD, with modulation of circulating glycosphingolipids and ganglioside species supporting their potential as pharmacodynamic biomarkers [37]. In parallel, oxysterol-derived bile acids have emerged as highly promising diagnostic markers. Elevated levels of 3β,5α,6β-trihydroxycholanic acid and its glycine conjugate, generated by abnormal cholesterol metabolism, were consistently detected in NPC plasma samples and can be accurately quantified in DBS, enabling highly sensitive and specific newborn screening strategies [39]. Additional NMR-based metabolomic studies identified alterations in plasma lipid-associated resonances, particularly triacylglycerols and amino acid signals such as isoleucine, distinguishing NPC1 patients from controls and heterozygous carriers and suggesting systemic metabolic reprogramming detectable in peripheral blood [40]. More recently, alkyl-LPCs have emerged as dynamic CSF biomarkers associated with treatment response and disease activity. Collectively, these findings support a multi-marker biomarker framework integrating lipid species, bile acid derivatives, and global metabolic signatures for NPC diagnosis, disease stratification, and therapeutic monitoring [38].

Untargeted metabolomic approaches combined with multivariate and pathway-level analyses have provided important insights into the systemic metabolic reprogramming associated with NPC. In cellular models of NPC1 deficiency, integrated LC–MS/MS analyses revealed widespread metabolic perturbations affecting multiple biochemical pathways, with progressive alterations observed in more severe NPC1 knockout conditions. Among the most consistently affected pathways were creatine and cysteine metabolism, indicating impaired energy homeostasis and altered redox regulation [41]. Complementary analyses of urinary NMR metabolomic datasets further identified coordinated disturbances in aromatic amino acid metabolism, including tryptophan–nicotinamide, kynurenine, histidine, and tyrosine pathways, together with reduced nicotinate and trigonelline levels, supporting disruption of interconnected amino acid and vitamin-related metabolic networks [42]. Collectively, these findings highlight NPC as a disorder characterised by interconnected alterations in amino acid metabolism, redox balance, and energy-related pathways, underscoring the value of systems-level metabolomics for identifying biologically coherent metabolic signatures not captured by conventional targeted approaches.

### 2.8. Acid Sphingomyelinase Deficiency

Acid Sphingomyelinase Deficiency (ASMD), also known as Niemann–Pick disease types A, B, and A/B, is a rare autosomal recessive disorder caused by pathogenic variants in the *SMPD1* gene. It is a multisystem disease characterised by progressive sphingomyelin accumulation in multiple organs, including the spleen, liver, lungs, bone marrow, and lymph nodes, with central nervous system involvement in the most severe forms. The clinical spectrum ranges from a rapidly progressive and fatal infantile neurovisceral form (type A) to intermediate neurovisceral (type A/B) and chronic visceral forms (type B), which show a slower course but remain associated with substantial morbidity and reduced life expectancy, particularly due to hepatic and respiratory complications. Disease rarity and phenotypic variability frequently lead to delayed diagnosis or misdiagnosis. Recently, ERT has been approved as a disease-modifying treatment, highlighting the importance of improving diagnostic pathways and clinical management strategies for affected patients [43,44,45].

Exhaled breath metabolomics has emerged as a promising non-invasive approach for the assessment of systemic metabolic alterations in lysosomal storage disorders. In adults with chronic visceral ASMD, GC–MS and LC–MS analysis of exhaled breath and breath condensate identified a distinct panel of volatile organic compounds (VOCs) that discriminated patients from healthy controls.

Among the most relevant compounds were 2-hydroperoxyhexane, 6-hepten-2-one, and 4-pentenyl acetate, which showed strong discriminatory performance and were not previously described in other pulmonary or systemic diseases. Several VOCs were associated with lipid peroxidation processes, suggesting a central role for oxidative stress and membrane lipid damage in shaping the exhaled metabolic profile. Multivariate analyses further demonstrated that VOC signatures could distinguish ASMD patients not only from controls, but also between subgroups with differing degrees of pulmonary involvement. Importantly, the metabolomic profiles correlated with clinical and functional parameters, including high-resolution computed tomography (HRCT) scores and diffusion capacity for carbon monoxide (DLCO), indicating a relationship between volatile metabolic alterations and disease severity. Overall, these observations support exhaled breath VOCs as a promising non-invasive biomarker class reflecting oxidative stress and lipid remodelling in ASMD, with potential applications in disease stratification and monitoring of pulmonary involvement [46].

**Figure 2:**
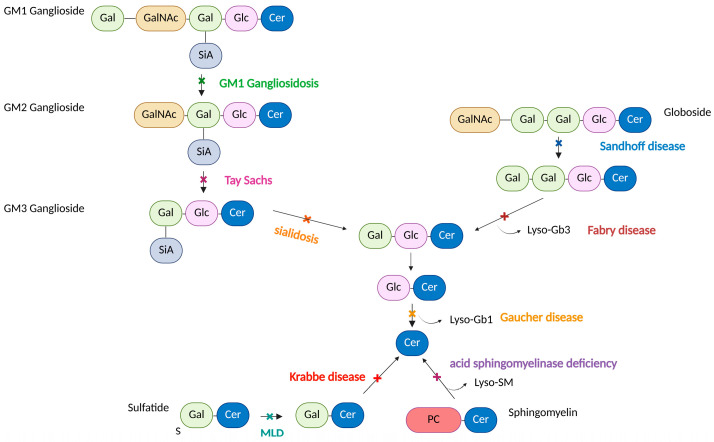
Schematic overview of the sphingolipidoses described in the previous section. For each LSD, the enzymatic defect underlying the disease is indicated by a cross. The enzymes involved in the pathway whose deficiency leads to the corresponding LSD are listed here. GM1 Gangliosidosis: GM1-β-galactosidase; Tay Sachs: β-hexosaminidase A; Fabry disease: α-galactosidase A; Gaucher disease: glucosylceramidase; Krabbe disease: β-galactosylceramidase; MLD, Metachromatic Leukodystrophy: arylsulfatase A; acid spingomyelinase deficiency: spingomyelinase. Gal: galactose; GalNAc: N-acetylgalactosamine; SiA: sialic acid; Glc: glucose, Cer: ceramide; PC: phosphatidylcholine. Created in https://BioRender.com.

Figure 2 and Table 1 summarise the key biochemical pathways and the main biomarkers unravelled with untargeted metabolomics approaches, offering an immediate vision of the sphinlipidoses described in this section.

## 3. Mucopolysaccharidoses

Mucopolysaccharidoses (MPS) represent a heterogeneous group of LSDs characterised by progressive intracellular deposition of glycosaminoglycans (GAGs) due to pathogenic variants in genes encoding lysosomal enzymes involved in their degradation. The resulting lysosomal dysfunction determines a wide range of cellular alterations (inflammation, oxidative stress, energy metabolism defects and tissue remodelling) which translate into a multisystem clinical picture of variable severity. Diagnosis is traditionally based on the quantification of urinary GAGs and the evaluation of specific enzymatic activity, but these approaches remain insufficient to predict progression, monitor therapeutic response and understand the pathobiological complexity of MPS [47,48]. In recent years, the advent of omics techniques, metabolomics, lipidomics, and proteomics, especially in untargeted modalities, has allowed the identification of new systemic and tissue-specific biomarkers, revealing previously unrecognised pathogenic pathways [49]. These studies have also highlighted how MPS profoundly involves global cellular metabolism and how alternative matrices to blood and urine (e.g., tissues, fibroblasts, murine models) can offer a more accurate perspective on the pathophysiology.

### 3.1. MPS I (Hurler, Hurler–Scheie, Scheie Syndrome)

MPS I is caused by mutations in the *IDUA* gene, responsible for the production of α-L-iduronidase. The enzyme is crucial for the catabolism of dermatan sulphate and heparan sulphate; Its deficiency causes lysosomal accumulation, impaired lysosomal function, inflammatory stress, and progressive tissue damage. Clinically, MPS I manifests across a spectrum of severity, from rapidly progressive forms with neurological involvement to more attenuated variants.

Quantification of urinary GAGs is the first diagnostic approach but has a poor correlation with clinical severity. More specific methods include measurement of non-reducing trisaccharides (NREs), which directly reflect the enzymatic defect. Highly sensitive LC-MS/MS techniques allow for the accurate detection of NREs, proving effective in monitoring response to ERT [50,51].

Untargeted LC-HRMS studies have revealed extensive metabolic remodelling, involving amino acids, carbohydrates, lipids, and metabolites associated with oxidative stress [49]. Proteomic analysis in mouse models has also highlighted marked dysfunction of the extracellular matrix, the inflammatory response, and fibrotic processes in the lung, demonstrating how the disease transcends the boundaries of classically studied tissues [52].

### 3.2. MPS II (Hunter Syndrome)

MPS II is caused by mutations in the *IDS* gene, transmitted in an X-linked manner, which results in a deficiency of the enzyme iduronate-2-sulfatase. The accumulation of heparan and dermatan sulphate generates a phenotype that varies between neuronopathic and non-neuronopathic forms.

Conventional biomarkers include urinary GAG measurement and enzyme activity, but they do not reliably predict progression, especially neurological progression [47,48].

Untargeted lipidomic analysis has identified gangliosides (GM2, GM3) and ceramides as potential new biomarkers, indicating a profound involvement of sphingolipid metabolism and a possible direct link to neurodegeneration [53]. In parallel, an exploratory metabolomics study on plasma and urine highlighted alterations in the histidine, tryptophan, and energy metabolism pathways, confirming a systemic metabolic imbalance [54]. These results reinforce the idea that assessment based solely on GAGs is insufficient and that a multi-omic panel approach may improve prognostic stratification [47,48].

### 3.3. MPS III (Sanfilippo Syndrome)

MPS III comprises four subtypes (A–D), each caused by a defect in a different enzyme involved in the catabolism of heparan sulphate. The clinical presentation is dominated by neurodegeneration, with cognitive regression, behavioural disturbances, sleep disturbances, and progressive motor impairment. Although urinary heparan sulphate measurement is diagnostic, its prognostic utility is limited [55]. In particular, the unpredictability of neurological progression makes the identification of predictive biomarkers of the disease course urgent.

Integrated metabolomic studies have shown complex alterations in membrane lipids, arginine pathways, oxidative stress, and mitochondrial bioenergetics [56]. Recent literature suggests interest in neurofilaments, sphingolipids, and inflammatory markers as possible indicators of brain progression, although clinical validation remains to be completed [55].

### 3.4. MPS IV (Morquio Syndrome)

MPS IV results from mutations in the *GALNS* (type A) or *GLB1* (type B) genes, which cause the accumulation of keratan sulphate. Unlike other MPSs, neurological involvement is rare; instead, severe skeletal manifestations (spondyloepiphyseal dysplasia, atlantoaxial instability) dominate. Quantification of urinary keratan sulphate is the primary diagnostic tool, but its correlation with clinical severity is modest [48] and reliable markers of skeletal progression are lacking.

Available studies are limited, and there are currently no untargeted metabolomic analyses dedicated exclusively to MPS IV. Some indirect evidence suggests alterations in inflammatory markers and extracellular matrix proteins [48], but more specific studies are needed.

### 3.5. MPS VI (Maroteaux–Lamy Syndrome)

MPS VI is caused by variants in the *ARSB* gene, which encodes arylsulfatase B, involved in the degradation of dermatan sulphate. The disease presents predominantly with variable severity of skeletal, cardiac, and respiratory phenotypes, without cognitive involvement. Urinary GAG assay remains the gold standard. However, interindividual variability limits the ability to accurately monitor progression.

An integrative metabolomics study has highlighted profound alterations in energy pathways, fatty acid metabolism, aromatic amino acid metabolism, and oxidative stress processes. An important contribution of this work is the combined use of plasma and cellular models, demonstrating the value of analysing unconventional matrices to understand processes that do not clearly emerge in traditional biological fluids [57].

### 3.6. MPS VII (Sly Syndrome)

MPS VII is caused by mutations in the *GUSB* gene, which encodes β-glucuronidase. The simultaneous accumulation of several GAGs (dermatan, heparan, and chondroitin sulphate) results in a highly variable phenotype: from perinatal hydropic forms to attenuated paediatric or adult presentations. Measurements of urinary GAGs and β-glucuronidase activity constitute the diagnostic reference, but they prove insufficient for assessing disease progression and the benefit of available therapies [58].

Unlike other MPS, no untargeted metabolomics studies dedicated to MPS VII are yet available, representing a significant gap in our knowledge of the pathogenic mechanisms and in the definition of emerging biomarkers [58].

**Figure 3:**
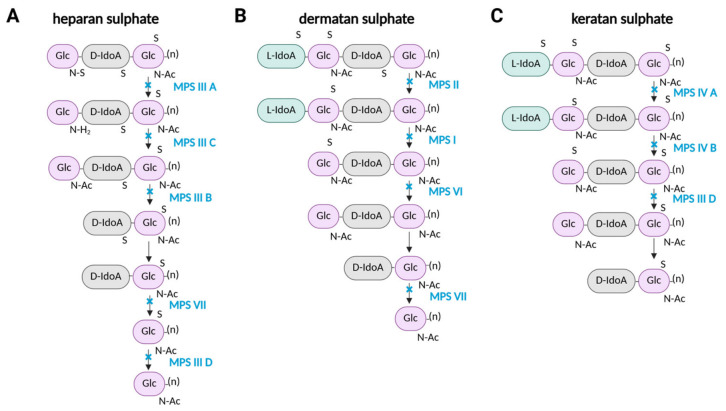
Schematic overview of the mucopolysaccaridoses described in the previous section. For each LSD, the enzymatic defect underlying the disease is indicated by a cross. Panel (**A**–**C**) represent the degradation of the main glycosaminoglycan chains, heparan sulphate, dermatan sulphate, and keratan sulphate, respectively. The enzymes involved in the metabolic pathway whose deficiency leads to the specific MPS are listed here. (**A**) MPS III A: heparan sulphatase; MPS III C: acetyl transferse; MPS III B: NAc glucosaminidase; MPS VII: β-glucoronidase; MPS III D: NAc glucosamine suplhatase; (**B**) MPS II: iduronate sulphatase; MPS I: iduronidase; MPS VI: NAc galactosamine suplhatase; MPS VII: β-glucoronidase; (**C**) MPS IV A: galactose-6-sulphatase; MPS IV B: β-galactosidase; MPS III D: NAc glucosamine sulphatase. Glc: glucose, IdoA: iduronic acid. Created in https://BioRender.com.

The key biochemical pathways of MPS are illustrated in Figure 3 highlighting the enzymatic steps of glycosaminoglycans degradation diving them in heparan sulphate, dermatan sulphate and keratan sulphate. Simultaneously, Table 2 summarises the potential biomarkers identified through untargeted metabolomics approaches. They bothprovide a metabolic photograph of the different MPS described previously.

## 4. Lysosomal Glycogen Storage Disease: Pompe Disease

Pompe Disease (PD), or Glycogen Storage Disease type II, is a rare autosomal recessive neuromuscular disease caused by mutations in the *GAA* gene, located on chromosome 17, which codes for the lysosomal enzyme acid α-glucosidase (GAA), essential for the degradation of glycogen in lysosomes [59,60]. The enzyme deficiency leads to progressive accumulation of glycogen in lysosomes, especially in muscle tissue, with consequent cellular damage and clinical manifestations ranging from severe infantile forms to progressively disabling late forms. For the clinical context, the disease presents a very heterogeneous spectrum that depends on the age of onset and the residual activity of the GAA enzyme, distinguishing infantile onset PD (IOPD) and the late onset PD (LOPD) forms [60,61].

The therapeutic approach includes ERT, it represents the current standard of care and has significantly improved the prognosis, especially in infantile forms [62]. However, ERT has limitations related to the immune response and the incomplete capacity to correct secondary cellular alterations [60,62]. Preclinical studies have shown that new generation therapies such as AT-GAA are more effective in reducing glycogen accumulation and improving autophagic and metabolic dysfunctions, acting more comprehensively on the AMPK/mTORC1 pathways [62]. These results highlight that correction of the primary defect alone is insufficient, emphasising the need to also target secondary cellular alterations [62]. Finally, the integration of genetics, metabolomics, proteomics and lipidomics has allowed to define PD as a complex multisystem pathology, in which the *GAA* gene defect triggers a network of interconnected metabolic and cellular dysfunctions. Overall, these findings highlight the pivotal role of multi-omic approaches in elucidating disease pathogenesis, identifying diagnostic biomarkers, and guiding the development of more effective and targeted therapeutic strategies [61].

From a pathophysiological point of view, PD is no longer considered a simple lysosomal storage disorder, but a complex condition characterised by a cascade of interconnected cellular alterations. Beyond the primary defect in glycogen degradation, disease pathogenesis involves autophagy dysfunction, alterations in the mechanistic target of rapamycin (mTOR) and AMP-activated protein kinase (AMPK) pathways, oxidative stress, calcium homeostasis imbalance, and mitochondrial dysfunction [61,62]. In experimental models, AMPK activation, reduced mammalian target of rapamycin (mTORC1) activity, accumulation of autophagosomes and ubiquitinated proteins are observed, highlighting a global alteration of muscle proteostasis [62]. Furthermore, a relevant role of mitophagy and key proteins such as Parkin, BNIP3 and NDP52 has been described, suggesting a direct involvement of mitochondrial turnover in the progression of muscle damage [63].

Another central element of pathogenesis is oxidative stress. Studies in stem cell-derived cardiomyocytes have demonstrated alterations in the NRF-2/ARE pathway and imbalances in cellular antioxidant systems, indicating impaired redox defence mechanisms that contribute to cardiac and muscle damage [64]. These processes are integrated with mitochondrial dysfunction and altered energy metabolism, suggesting a key role for oxidative stress in the systemic pathology of PD [64].

Multi-omic studies further expanded the understanding of the molecular mechanisms of the disease. Proteomic and lipidomic analyses highlighted increased expression of glycolytic enzymes, including lactate dehydrogenase B (LDHB) and pyruvate kinase M (PKM), alongside reduced levels of proteins involved in phospholipid metabolism, such as glycosylphosphatidylinositol-specific phospholipase D (GPLD1) and paraoxonase 1 (PON1), suggesting marked dysregulation of glucose and lipid metabolism [61,62]. These changes are associated with dysfunctions of autophagy, inflammation, calcium homeostasis and antioxidant response, outlining a complex systemic picture [61,62,64].

In a metabolomic framework, the disease is characterised by important alterations in energy metabolism. In muscle tissues and cellular models, decreased glycolytic metabolites together with increased Krebs cycle intermediates and fatty acids suggest a metabolic shift toward lipid β-oxidation as an alternative energy source. Collectively, these findings indicate that PD is not merely a storage disorder, but also a disease characterised by impaired cellular energy homeostasis [62].

Transcriptomic and proteomic analyses in LOPD forms have highlighted alterations of the lysosomal pathway, increased glycolysis, dysfunction of oxidative phosphorylation, impaired mitophagy and alterations of calcium homeostasis [61]. In particular, the integrated study of proteomics and metabolomics also identified potential biomarkers such as calmodulin-like protein 3 (CALML3) and neurofilament light polypeptide (NEFL), suggesting a multisystem involvement of the disease [63].

A central diagnostic role is played by urinary metabolic biomarkers, in particular the glycogen-derived tetrasaccharide Glc4, which is significantly increased in patients and is also used for therapeutic monitoring [60,61]. More recent studies have also highlighted the diagnostic value of other oligosaccharides such as Hex5, Hex6 and Hex7, especially in combination in multi-metabolite panels with high diagnostic accuracy [61]).

**Figure 4:**
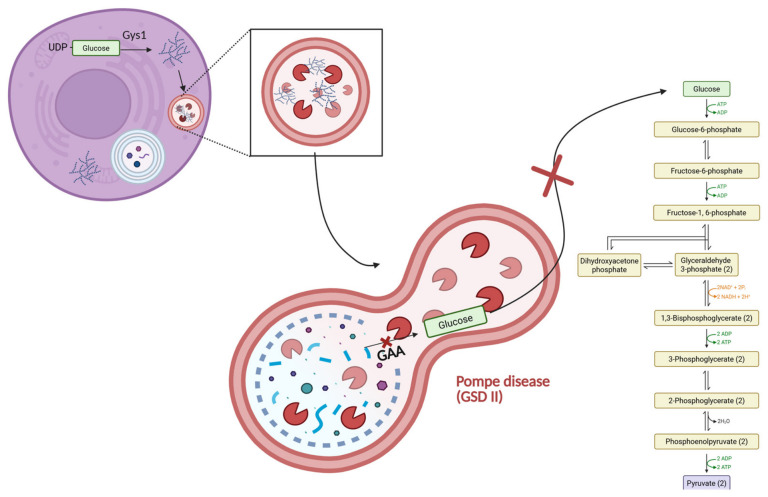
The figure illustrates the massive accumulation of glycogen within lysosomes in Pompe disease, resulting from the loss of lysosomal acid α-glucosidase (GAA) activity. GAA catalyses the hydrolysis of glycogen to glucose within the lysosome; therefore, its deficiency leads to progressive glycogen storage, lysosomal enlargement, and lysosomal dysfunction. Created in https://BioRender.com.

Table 3 and Figure 4 provide an immediate overview of Pompe disease underling the *GAA* deficiency and the potential biomarkers revealed by untargeted metabolomics studies.

## 5. Oligosaccharidoses

Oligosaccharidoses, or glycoproteinoses, are inherited lysosomal storage disorders caused by defects in enzymes involved in glycoprotein degradation, leading to intracellular accumulation of undegraded oligosaccharides and their urinary excretion. Clinical manifestations are heterogeneous and often include facial dysmorphism and progressive neurological deterioration [65]. Characteristic urinary oligosaccharide patterns have been described for several subtypes, including mannosidosis, fucosidosis, aspartylglucosaminuria, GM1 gangliosidosis, sialidosis, and galactosialidosis, mainly identified through MS–based analyses [66].

A-Mannosidosis is caused by mutations in the *MAN2B1* gene, leading to α-mannosidase deficiency and accumulation of mannose-containing oligosaccharides. Clinical manifestations include psychomotor delay, hearing loss, skeletal abnormalities, and progressive neurodegeneration; ERT with velmanase alfa can reduce oligosaccharide accumulation but has limited neurological efficacy [67]. B-Mannosidosis, due to *MANBA* mutations and β-mannosidase deficiency, is an ultra-rare disorder characterised by developmental delay, intellectual disability, behavioural abnormalities, and hearing loss, with diagnosis based on oligosaccharideuria and MS findings [68]. Fucosidosis results from *FUCA1* mutations causing α-L-fucosidase deficiency, with accumulation of fucosylated substrates leading to neurodegeneration, cognitive decline, angiokeratomas, hepatosplenomegaly, and skeletal abnormalities; no curative therapy is currently available [69]. Sialidosis is associated with *NEU1* mutations and neuraminidase deficiency, causing accumulation of sialylated oligosaccharides. Clinical severity ranges from mild type I forms with myoclonus and ataxia to severe type II forms with dysmorphism, intellectual disability, and progressive neurodegeneration [70]. Galactosialidosis, caused by *CTSA* mutations, leads to combined deficiency of neuraminidase and β-galactosidase, resulting in lysosomal accumulation of glycoproteins and oligosaccharides with systemic and neurological involvement [71]. Aspartylglucosaminury, due to *AGA* mutations and glycosylasparaginase deficiency, is characterised by progressive cognitive decline, motor impairment, and accumulation of glycoasparagine and related glycopeptides [72].

Although these disorders are characterised by distinct urinary oligosaccharide profiles, the available literature mainly relies on targeted biochemical and MS analyses. As a result, untargeted metabolomics has yet to be extensively applied to these disorders, leaving important gaps in the understanding of their global metabolic landscape and limiting opportunities for the discovery of novel biomarkers and therapeutic targets.

**Figure 5:**
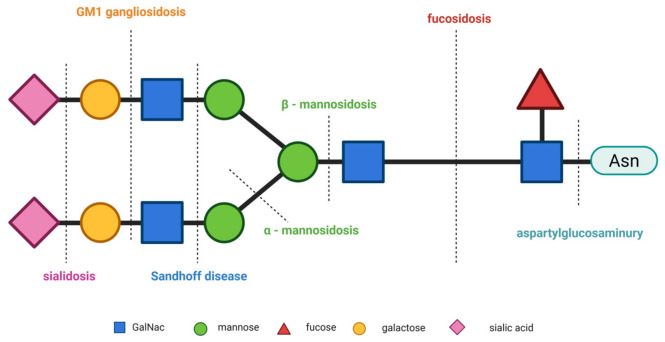
The figure schematically illustrates the major oligosaccharidoses and the corresponding lysosomal degradation pathways. Dotted lines indicate the specific step at which the deficient lysosomal enzyme acts, highlighting the site of the metabolic block responsible for impaired oligosaccharide catabolism. Legend shapes and colours is provided in the figure. Created in https://BioRender.com.

The principal results in terms of potential biomarkers unravelled by metabolomics approaches are listed in Table 4. Instead, Figure 5 provides a schematic overview of the different oligosaccharidosis, highlighting the specific lysosomal enzyme deficiency underlying each disorder. For each disease, the figure facilitates the understanding of the molecular basis of each oligosaccharidosis by indicating the precise step in the oligosaccharide degradation pathway at which the enzymatic defect occurs.

## 6. Lysosomal Proteinoses

Lysosomal proteinoses are a subgroup of LSDs caused by impaired lysosomal protein degradation or transport, leading to intracellular accumulation and cellular dysfunction.

### 6.1. Cystinosis

Cystinosis is a rare, autosomal recessive, monogenic metabolic disorder caused by homozygous or compound heterozygous pathogenic variants in the *CTNS* gene, which encodes cystinosin, a ubiquitously expressed lysosomal cystine transporter [73]. The estimated incidence of cystinosis is 1 in 100,000–200,000 live births [74]. Loss of cystinosin function leads to the progressive intralysosomal accumulation of cystine, with the kidneys being the first and most severely affected organs. Over time, cystine crystal deposition extends to multiple tissues, including the cornea, endocrine and reproductive systems, muscles, bones, lungs, skin, and the central nervous system.

Beyond lysosomal cystine accumulation, the pathogenesis of cystinosis is increasingly recognised as multifactorial and incompletely understood. It involves dysregulation of several interconnected cellular pathways, including inflammatory and fibrotic signalling, autophagy, mTOR signalling, lysosomal biogenesis, and vesicular trafficking. These alterations likely contribute to progressive cellular dysfunction and tissue injury, indicating that cystine accumulation alone is insufficient to fully explain disease severity and clinical progression [75].

Clinically, cystinosis comprises three major phenotypes: infantile nephropathic, juvenile nephropathic, and adult non-nephropathic (ocular) forms. The infantile form is the most common and severe, typically presenting during the first year of life with renal Fanconi syndrome and progressive chronic kidney disease; the juvenile form exhibits later onset and milder renal manifestations, whereas the adult form is predominantly characterised by corneal cystine crystal deposition and photophobia with limited systemic involvement [76]. Diagnosis is based primarily on elevated leukocyte cystine concentrations, supported by molecular analysis of *CTNS* and ophthalmologic identification of corneal crystals. Currently, cysteamine remains the only disease-specific therapy, reducing lysosomal cystine accumulation and delaying disease progression, although renal dysfunction and Fanconi syndrome frequently persist despite treatment, highlighting the need for a deeper understanding of disease mechanisms and novel therapeutic strategies [73].

In this context, omics-based approaches have provided important insights into the systemic metabolic consequences of *CTNS* deficiency. Using an in vitro HK-2 cell model with *CTNS* silencing, Baysal et al., performed an integrated multi-omics analysis combining metabolomics, fluxomics, and proteomics, demonstrating profound metabolic reprogramming beyond cystine storage. Metabolomic profiling revealed significant reductions in metabolites involved in amino acid, sulphur, glutathione, and energy metabolism, particularly affecting alanine, aspartate, glutamate, arginine, methionine, and glutathione pathways, consistent with impaired redox homeostasis and compensatory cysteine synthesis. Fluxomic analyses showed increased turnover of Krebs cycle intermediates, including fumarate, malate, and citrate, suggesting altered mitochondrial activity and energy metabolism, while proteomic data identified dysregulation of proteins involved in apoptosis, ribosome biogenesis, and protein synthesis, supporting the role of oxidative stress and proteostatic imbalance in disease progression. Altogether, these integrated omics findings expand the current understanding of cystinosis pathophysiology, identifying altered metabolic and mitochondrial pathways as potential biomarkers and therapeutic targets beyond lysosomal cystine accumulation alone [77].

### 6.2. Neuronal Ceroid Lipofuscinosis

Neuronal Ceroid Lipofuscinoses (NCLs), or Batten diseases, represent one of the most common groups of rare, inherited neurodegenerative LSD in childhood. The global prevalence of NCL varies depending on geographic region and specific genetic subtype, with estimates ranging from 0.01 to 9 cases per 100,000 live births. NCLs are a genetically heterogeneous group of lysosomal storage neurodegenerative disorders caused by mutations in at least 14 genes (*CLN1*–*CLN14*), encoding proteins with diverse subcellular localizations and functions but converging on lysosomal dysfunction. *CLN1* and *CLN2* encode the soluble lysosomal hydrolases palmitoyl-protein thioesterase 1 (PPT1) and tripeptidyl peptidase 1 (TPP1), whose deficiency impairs the degradation of lipid-modified proteins and peptides. *CLN3* encodes a lysosomal/endosomal transmembrane protein involved in intracellular trafficking, while *CLN5* encodes a soluble lysosomal glycoprotein of still incompletely defined function. *CLN6*, *CLN7* (*MFSD8*), and *CLN8* encode membrane-associated proteins of the endoplasmic reticulum and endolysosomal system that are implicated in protein trafficking and lysosomal biogenesis. *CLN10* corresponds to cathepsin D, a lysosomal protease, whereas *CLN11* (progranulin) is involved in lysosomal function and neuroinflammatory regulation. *CLN12* encodes a lysosomal cation transporter, *CLN13* (cathepsin F) a lysosomal protease, and *CLN14* a protein implicated in neuronal excitability and survival. Although the primary gene products differ in localization and function, most NCL subtypes share the pathological hallmark of progressive lysosomal storage dysfunction with accumulation of autofluorescent material, frequently including subunit c of mitochondrial ATP synthase. The mechanisms linking these primary defects to selective vulnerability of neurons and retinal cells remain incompletely defined [78].

In this context, untargeted metabolomics has emerged as a promising approach for investigating global biochemical alterations associated with disease pathogenesis and for identifying candidate biomarkers, although its application in NCLs remains limited, primarily to *CLN2* and *CLN6* disease.

*CLN2* disease is a rare autosomal recessive LSD caused by pathogenic variants in the *TPP1* gene, which encodes the lysosomal enzyme tripeptidyl peptidase 1. Loss of TPP1 activity results in progressive accumulation of lipofuscin within neurons and other cell types, ultimately leading to widespread neurodegeneration characterised by epilepsy, motor and cognitive decline, and premature death [79]. Although the genetic and pathological basis of *CLN2* disease is well established, the metabolic consequences of TPP1 deficiency remain poorly understood. In this regard, Sindelar et al. applied untargeted LC–MS-based metabolomic profiling of CSF from patients with *CLN2* disease and identified a robust and reproducible metabolic signature associated with both clinical severity, assessed using the Weill Cornell LINCL Scale (WCLS), and MRI-based disease burden measured by the Magnetic Resonance Imaging Disease Severity Score (MRIDSS). Specifically, eight metabolites were consistently reduced in CLN2 CSF, predominantly acetylated compounds, including N-acetylaspartylglutamate (NAAG), N-acetylneuraminic acid and its dimer, N-acetylalanine, N-acetylserine, N-acetylthreonine, glycerol-3-phosphoinositol, and sulfoacetic acid. Notably, these metabolites showed linear associations with disease progression, suggesting a potential link with the underlying neurodegenerative process [80].

Collectively, these findings define a characteristic *CLN2*-associated CSF metabolic signature and support the use of untargeted metabolomics for biomarker discovery in lysosomal storage disorders, although further validation in independent and longitudinal cohorts is required. Similarly, untargeted metabolomics also provided important insights into *CLN6* disease, an autosomal recessive disorder caused by mutations in the *CLN6* gene, whose encoded protein still lacks a fully defined biological function. Affected individuals typically present between 18 months and 8 years of age, with early clinical manifestations including ataxia, seizures, and progressive cognitive decline [81].

Given the limited understanding of CLN6 pathogenic mechanisms and the lack of effective therapies [82], Corina-Marcela Rus et al. performed the first untargeted LC–MS-based metabolomic analysis in human *CLN6*-derived neuronal cells generated through reprogramming of patient fibroblasts into induced neuronal progenitor cells (ciNPCs). Their study identified 15 significantly downregulated metabolites with strong discriminatory power between *CLN6* and healthy controls. Among these, five metabolites with variable importance in projection (VIP) scores > 1, PG 40:6, PG 40:7, C16 GlcCer, C24 GlcCer, and C24:1 GlcCer, emerged as the major contributors to group separation, highlighting profound dysregulation of lipid metabolism. Pathway analysis further demonstrated prominent alterations in sphingolipid and glycerophospholipid metabolism, suggesting a central role for these lipid pathways in *CLN6* pathogenesis. In particular, reduced glucosylceramide levels, together with alterations in metabolites such as cyclic ADP-ribose and vitamin K1, proposing a potential impairment of calcium signalling pathways. Overall, these findings demonstrate substantial alterations in lipid metabolism and cellular signalling in *CLN6* disease and further support the relevance of untargeted metabolomics not only for biomarker identification but also for elucidating the molecular mechanisms underlying NCLs [83].

### 6.3. Danon Disease

Danon Disease (DD) is a rare X-linked multisystem disorder caused by mutations in the *LAMP2* gene, which encodes lysosome-associated membrane protein 2 (LAMP-2), a key regulator of autophagy [84]. LAMP-2 is essential for lysosomal function, particularly in mediating the fusion of autophagosomes with lysosomes and regulating autophagic flux. Among patients with hypertrophic cardiomyopathy (which occurs in 1: 500 individual), pathogenic *LAMP2* variants have been identified in about 1–4% of cases. Impairment of this pathway leads to the accumulation of autophagic vacuoles in cardiac and skeletal muscle cells, representing a hallmark pathological feature of the disease. Clinically, DD is characterised by cardiomyopathy, skeletal myopathy, and variable degrees of cognitive impairment. The *LAMP2* gene encodes three isoforms (LAMP-2A, LAMP-2B, and LAMP-2C), which differ in their structural domains, tissue distribution, and functional roles in autophagy. LAMP-2A is ubiquitously expressed and is essential for chaperone-mediated autophagy, whereas LAMP-2B is predominantly expressed in cardiac and skeletal muscle and in the brain, suggesting a central role in tissue-specific disease pathogenesis. LAMP-2C is less well characterised but has been implicated in nucleic acid degradation pathways. While most pathogenic variants result in loss of all three isoforms, mutations selectively affecting. LAMP-2B has also been reported, indicating that its deficiency may be sufficient to drive disease pathogenesis and highlighting it as a potential therapeutic target. DD exhibits marked sex-dependent differences. Male patients usually present in childhood or adolescence with the classical triad of severe hypertrophic cardiomyopathy, skeletal myopathy, and mild cognitive impairment, often associated with rapid cardiac progression, arrhythmias, and early heart failure requiring transplantation. In contrast, females generally develop a later-onset and more heterogeneous phenotype, predominantly characterised by hypertrophic or dilated cardiomyopathy, conduction abnormalities, and arrhythmias, while skeletal muscle and cognitive involvement are typically mild or absent. Additional manifestations in both sexes may include retinopathy, peripheral neuropathy, hepatomegaly, and gastrointestinal dysfunction [85]. Diagnosis is based on clinical presentation, cardiac imaging, and confirmation of pathogenic *LAMP2* variants by molecular genetic testing, while muscle biopsy showing vacuolated myocytes with autophagic material accumulation is now less frequently required. Currently, no disease-specific therapy is available, and management remains supportive, focusing on heart failure treatment, arrhythmia control, prevention of sudden cardiac death, and heart transplantation in advanced stages [86].

Recent multi-omics studies have significantly expanded the understanding of DD pathogenesis by integrating transcriptomic and untargeted metabolomic analyses in patient-derived cardiac tissue, hiPSC-cardiomyocytes, and fibroblasts. These investigations revealed profound metabolic reprogramming associated with impaired autophagy and mitochondrial dysfunction, characterised by a shift toward glycolytic metabolism with accumulation of glycolytic and TCA cycle intermediates, increased L-carnitine and propionyl-carnitine in patient-derived hiPSC-cardiomyocytes (although not confirmed in cardiac tissue), alterations in NAD^+^ metabolism, and reduced oxidative phosphorylation efficiency, suggesting activation of compensatory metabolic routes. Transcriptomic analyses further demonstrated signatures of mitochondrial impairment, inflammation, and extracellular matrix remodelling associated with fibrosis and arrhythmogenesis. Altogether, these findings support the existence of a Warburg-like metabolic adaptation in DD, linking lysosomal dysfunction to altered cardiac energy homeostasis and progressive myocardial remodelling, while highlighting the potential of integrated omics approaches for biomarker discovery and identification of novel therapeutic targets [87].

### 6.4. Lysosomal Cathepsin Deficiencies

Lysosomal Cathepsins (CTSs) constitute a major family of proteolytic hydrolases essential for intracellular protein degradation and maintenance of lysosomal homeostasis. Together with the proteasome, the lysosomal compartment represents one of the principal cellular proteolytic systems and plays a critical role in amino acid recycling, particularly under conditions of starvation and autophagy. Based on their catalytic mechanism, CTSs are classified into aspartic, cysteine, and serine proteases, and are involved in several physiological processes including autophagy, innate immunity, apoptosis, and protein turnover. Deficiencies of lysosomal CTSs have been associated with severe neurodegenerative alterations and intracellular accumulation of undegraded material resulting from impaired autophagic flux and lysosomal dysfunction. Although proteostasis impairment and lysosomal alterations associated with CTS defects have been investigated through cellular and proteomic approaches, untargeted metabolomic studies specifically focused on lysosomal CTS deficiencies are still lacking [88]. Together with other omics approach untargeted metabolomic investigations could contribute to the identification of novel biomarkers and the elucidation of disease-specific metabolic pathways.

**Figure 6:**
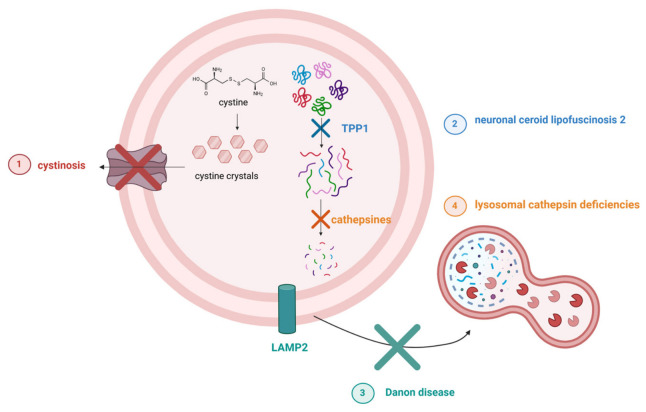
The figure shows a lysosome where the main lysosomal proteinoses are described in their biochemical pathways: (1) cystinosis where the lysosomal cystine transporter is blocked causing an accumulation of cystine crystals; (2) neuronal ceroid lipofuscinosis 2 caused by mutations in the *TPP1* gene, which result in a deficiency of the tripeptidyl peptidase 1 enzyme; (3) Danon disease caused by mutations in the *LAMP2* gene, which encodes lysosome-associated membrane protein 2 (LAMP-2), a key regulator of autophagy; (4) lysosomal cathepsin deficiencies where cathepsins, a family of protease enzymes, are the key responsible for amino acid recycling, especially under conditions of starvation and autophagy. Created in https://BioRender.com.

Figure 6 provides a schematic overview of the major lysosomal proteinoses discussed in this section. The figure depicts a representative lysosome and illustrates the specific metabolic pathways affected in each disorder, highlighting the enzymatic deficiency and the corresponding metabolic blocks. Together with Table 5 it is an impressive photograph of this LSDs class. In particular, metabolites found by untargeted metabolomics analyses and studies are described as putative candidate biomarkers for lysosomal proteinoses in Table 5.

## 7. Mucolipidoses

Mucolipidoses (ML) are rare autosomal recessive LSD caused by deficiency of UDP-N-acetylglucosamine-1-phosphotransferase (GlcNAc-PTase), the enzyme responsible for mannose-6-phosphate (M6P) tagging and correct lysosomal targeting of acid hydrolases. In the absence of M6P, lysosomal enzymes fail to reach the lysosome, resulting in intracellular accumulation of undegraded substrates. The worldwide incidence is estimated at 2.5–10 cases per million live births [89,90].

GlcNAc-1-phosphotransferase is a heterohexameric enzyme composed of α, β, and γ subunits (α_2_β_2_γ_2_). The α and β subunits are encoded by the *GNPTAB* gene, and pathogenic variants in this gene cause ML II α/β and ML IIIA, whereas variants in *GNPTG*, encoding the γ subunit, are associated with ML IIIC [90,91]. Functional studies demonstrated absent or severely reduced enzyme activity in *GNPTAB*-mutant fibroblasts, indicating a major catalytic defect. In contrast, *GNPTG*-mutant fibroblasts retain normal activity toward simple sugar substrates but show impaired phosphorylation of acid hydrolases, supporting a role of the γ subunit in protein substrate recognition. Based on clinical severity and disease progression, ML is classified into ML II and ML III, with ML III representing a milder attenuated form characterised by slower progression and longer life expectancy [92].

Patients with ML II and ML III show clinical overlap with other lysosomal storage disorders, particularly MPS, making differential diagnosis challenging. Although direct measurement of UDP-GlcNAc-1-phosphotransferase activity represents the most reliable diagnostic approach, the assay is technically demanding and not widely available. Consequently, diagnosis largely relies on combined biochemical and molecular genetic investigations, while defective GlcNAc-1-phosphotransferase activity results in mis-sorting of lysosomal enzymes, leading to increased lysosomal enzyme activities detectable in plasma, DBS, and culture media from fibroblasts or amniocytes. In addition, biochemical assays based on mannose-6-phosphate (M6P)-specific antibody recognition have been developed to distinguish ML from MPS.

However, lysosomal enzyme activity profiles and M6P-containing protein levels do not reliably differentiate ML II from ML III, nor ML III α/β from ML III γ. Therefore, definitive diagnosis requires sequencing of *GNPTAB* and *GNPTG* [92].

Despite the growing application of untargeted metabolomics across LSDs, comparable studies in ML remain lacking, leaving the systemic metabolic consequences of defective lysosomal enzyme trafficking largely unexplored.

### 7.1. ML II (Mucolipidosis Type II)

ML II represents the most severe phenotype within the ML spectrum and is a multisystem disorder that may already manifest prenatally, including cases of hydrops fetalis. The disease is primarily characterised by dysostosis multiplex, resulting in profound growth restriction with short stature and low body weight. Affected individuals typically present with coarse facial features, developmental delay, progressive joint contractures, marked gingival hyperplasia, hepatosplenomegaly, abdominal wall defects, hypotonia, and recurrent respiratory infections.

Cardiovascular involvement and respiratory complications are common and are largely associated with mucosal thickening and thoracic cage rigidity. Early postnatal secondary hyperparathyroidism has also been frequently reported. Unlike many other LSDs, ML II often presents with clinical manifestations evident at birth, and the phenotype is frequently distinctive enough to support early diagnostic suspicion. In addition, prenatal radiological abnormalities have been described, facilitating early recognition [92]. Collectively, these severe multisystem manifestations lead to profound motor and cognitive impairment, generally resulting in death during early childhood [93].

### 7.2. ML III (Mucolipidosis Type III)

ML III exhibits a broad phenotypic spectrum, ranging from severe childhood-onset forms, often considered intermediate ML II/III α/β phenotypes, to milder presentations compatible with survival into late adulthood. Among ML subtypes, ML III γ generally displays the mildest clinical course [93]. Currently, no curative therapy is available for ML, and management remains primarily supportive and symptomatic.

**Figure 7:**
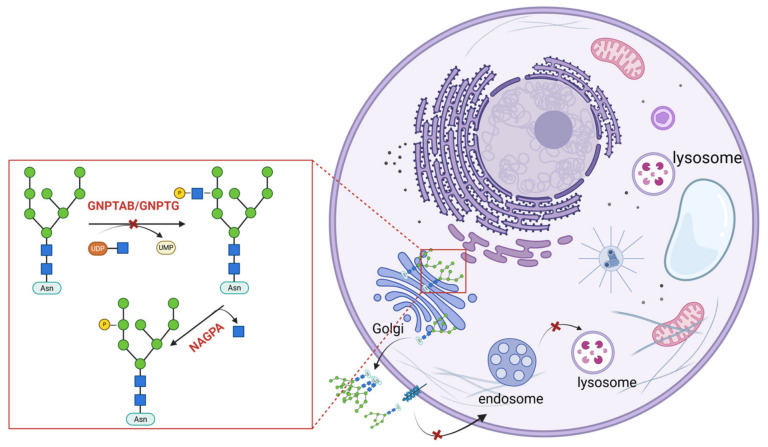
The figure shows an example of cells of MLII- and MLIII-affected individuals where the correct incorporation of the phosphate—catalysed by the enzyme encoded by the *GNPTAB* and *GNPTG* genes—is impaired. Under physiological conditions, lysosomal soluble enzymes are produced in the endoplasmic reticulum and subsequently delivered to the Golgi apparatus, where they receive mannose-6-phosphate (M6P) tags through the action of GlcNAc-1-phosphotransferase. Then, the M6P-modified lysosomal enzymes are recognised by M6P receptors and transported via endosome to lysosomes. Instead, MLII- and MLIII cells defect in GlcNAc-1-phosphotransferase creating a messy and hypersecretion of lysosomal enzymes into the extracellular space. As shown in the figure, the first reaction is catalysed by GlcNAc-1-phosphotransferase and the substrate is UDP-GlcNAc; then, the GlcNAc is removed in the second reaction catalysed by N-acetylglucosamine-1-phosphodiester α-N-acetylglucosaminidase encoded by *NAGPA* gene, leaving the M6P residue alone. GlcNAc: *N*-acetylglucosamine; M6P: mannose 6-phosphate. Green circles represent manjose residues, instead blue squares GlcNAc residues. Created in https://BioRender.com.

M6P-dependent trafficking of lysosomal enzymes was schematized in Figure 7, where a representative animal cell is depicted by illustrating the main actors of M6P trafficking performed by lysosomal enzymes. Simultaneously Table 6 provides a summary of key alterations and potential biomarkers referred to MLII- and MLIII. In this regard, despite substantial advances in understanding the molecular and cellular mechanisms underlying mucolipidoses, untargeted metabolomic investigations remain very limited. As a result, the systemic metabolic perturbations associated with these disorders have yet to be comprehensively characterised, restricting the identification of novel biomarker candidates and the elucidation of disease-related metabolic pathways by exploiting the combination of multi-omics approaches.

## 8. Emerging Technologies for Untargeted Metabolomics in LSDs

### 8.1. Early Targeted Diagnostic Technologies for LSDs

The earliest approaches for the assessment of LSDs almost exclusively relied on targeted biochemical assays that measured the activity of single enzymes known to be deficient in specific disorders. The earliest biochemical assays that identified LSDs were first reported in 1963, which identified α-glucosidase deficiency in PD [94] and ASA deficiency in MLD [95]. As both the understanding of metabolic pathways improved and technology evolved, fluorometry using artificial fluorescent substrates, such as 4-methylumbelliferyl (4-MU), became a standard method for targeting enzymatic activity across a range of LSDs. Capable of providing greater levels of sensitivity for detecting changes in enzyme activity in LSDs, fluorometry still remains a cornerstone in clinical laboratories, particularly for assessing DBS in newborn screening, and are known to be a reliable approach for LSD enzyme measurement, covering disorders such as Pompe [96], Gaucher [97], Fabry [98], and MPS [99]. Recent advances in this technology have led to a digital microfluidics (DMF) device that can measure these LSDs as part of a first-tier testing strategy [99] (Figure 1).

### 8.2. Transition to Larger Panels and Workflows for LSDs

The introduction and growing availability of tandem mass spectrometry (MS/MS), alongside the implementation of soft ionisation techniques such as Electrospray Ionisation (ESI) [100], marked an important step towards both targeted and untargeted metabolomic workflows. ESI provided a conduit to couple liquid chromatography to MS, both simplifying sample introduction and enabling soft ionisation of analytes directly from the liquid phase. In addition, by combining two mass-filtering quadrupoles with a collision cell, MS/MS instruments isolate precursor ions, fragment them in a controlled manner, and detect the resulting product ions. Because analytes generate characteristic fragmentation patterns, this configuration supports selective, simultaneous quantification of metabolites, lipids, and enzyme substrates. Flow injection analysis (FIA) coupled to MS/MS was developed as a technique to investigate a range of LSDs in a multiplexed assay [101], and this was later expanded to the application of LC-MS/MS, implementing chromatographic separation to provide greater selectivity in measuring the activity of nine lysosomal enzymes while maintaining a workflow and runtime that is compatible with NBS laboratories [102]. These targeted LC-MS/MS methods have continued to evolve in their scope and complexity, with a single assay capable of targeting 19 s-tier biomarkers for NBS, including PD [103].

Though the expansion of analytical capabilities has assisted with the diagnosis of LSDs, there are limitations of targeted metabolomic approaches that cannot be overcome. LSD biomarkers are not always specific; urinary glucose tetrasaccharide (Glc4), an established biomarker for PD, shows elevated concentrations in other glycogen storage disorders [104], underscoring the need for complementary biomarkers to increase confidence in diagnosis. This clinical heterogeneity is a challenge, as many LSDs show variable or subtle phenotypes across the same disorder, often seen in heterozygous populations, as well as overlap between the presentation of different LSD subtypes, requiring further biochemical and genetic testing for confirmation. Pseudo-enzymatic deficiencies highlight the need to expand analytical capabilities, exemplified by ASA activity in MLD, where low ARSA activity can be observed alongside normal urinary sulfatides and neurological symptoms, illustrating that low in vitro enzymatic activity does not equal disease [105]. To help address some of these subtle changes, having a greater understanding of the role of lysosomal biology in a wider context is key, meaning untargeted approaches can offer insight into cell-wide disturbances caused by LSDs, including in autophagy, trafficking, and signalling [106].

### 8.3. Untargeted Techniques for Metabolomic Profiling of LSDs

The emergence of user-friendly hybrid High-Resolution Mass Spectrometry (HRMS) platforms, such as Orbitrap and Quadrupole Time-of-Flight (QTOF) systems, provided an avenue for exploring a broader, untargeted view of lysosomal biology using MS techniques. While both platforms measure analytes by mass-to-charge (*m*/*z*) ratios, the separation principles of these HRMS systems differ: Orbitrap analysers discriminate ions by their axial oscillation frequencies within an electrostatic field [107], whereas TOF instruments separate ions by their flight time to the detector [108]. More recent advances in HRMS systems incorporate ion mobility (IM) [109], which provide an additional orthogonal separation mechanism; first separation of analytes in the gas phase by how quickly they drift through a neutral gas under an electric field, which reflects their size, shape, and charge, followed by further resolution of the analytes based on their mass-to-charge ratio (*m*/*z*). The ultra-high mass resolution and low-ppm mass accuracy of HRMS exceed the capabilities of unit-resolution MS/MS systems, used in targeted NBS applications, enabling confident identification of molecular species across wide dynamic ranges. When coupled with chromatographic separation, HRMS provides excellent selectivity and sensitivity and can support both untargeted approaches to identify potential biomarker panels, followed by translation of these biomarkers into targeted metabolomic workflows for assessing LSDs in a diagnostic setting. Untargeted analysis with LC-IM-HRMS revealed dysregulation in amino acid metabolism in MPS VI patients, with targeted LC-MS/MS analysis of amino acids and acylcarnitines corroborating the extent of the dysregulation observed in these pathways [57]. Glycomic profiling of urine samples with LC-IM-HRMS for untargeted biomarker discovery revealed distinct glycans as new biomarkers for identifying subtypes of MPS [110], while another untargeted study using LC-HRMS noted increased concentrations of dipeptides, amino acids, and derivatives in the MPS group relative to controls [49]. Combining untargeted and targeted LC-HRMS metabolomics analysis also revealed complementary biomarkers to Glc4 in PD, thereby increasing the specificity of sample-class prediction compared with using a single Glc4 biomarker [111]. Combined approaches have been adopted to identify and evaluate potential new biomarkers for Niemann-Pick Type C [41] and key sulfatide species for the diagnosis of MLD [112]. An alternative, rapid approach for untargeted HRMS analysis, Matrix-Assisted Laser Desorption Ionisation (MALDI), is a soft ionisation technique used in MS to analyze large and fragile molecules, such as proteins, peptides, lipids, and oligosaccharides [113]. It allows direct analysis of samples and, when coupled to TOF MS, addresses some of the limitations of Thin Layer Chromatography (TLC), typically used for the diagnosis of Oligosaccharidoses. MALDI TOF-MS can deliver rapid and specific pattern-based molecular fingerprints of free oligosaccharides (FOCs) in urine, providing a tool for screening oligosaccharidosis and other LSDs such as Pompe and GD [114].

Since its first published use for metabolic profiling in 1974 [115], Nuclear Magnetic Resonance (NMR) spectroscopy, like HRMS, has become an established platform for metabolomic studies, and can complement HRMS in untargeted analysis of LSDs. NMR is based on aligning atomic nuclei with a magnetic field, exciting them with radiofrequency pulses, and measuring the emission signals from the nuclei as they relax back to their lower-energy state. The resulting spectrum can reveal details about the molecular structure and dynamics of the analytes [116]. The technique can be applied to non-invasive studies, requiring minimal sample preparation and high reproducibility, giving researchers insights into LSDs pathology and treatment. NMR techniques include solution-state NMR, high-resolution Magic Angle Spinning (HR-MAS) NMR, and in-cell NMR. Solution-state NMR is a widely used classic NMR spectroscopy approach for measuring molecules in biological fluids and encompasses applications targeting protons (1H NMR), carbon isotopes (13C NMR), and phosphorus (31P NMR). 1H-NMR, a method that monitors the behaviour of protons in a magnetic field, has been applied in untargeted metabolomics to reveal additional biomarkers related to energy demand metabolic pathways in patients with GM1 Type 2 Gangliosidosis [32], offering the option of monitoring global metabolic signatures rather than relying solely on targeted biomarkers such as β-galactosidase and neuraminidase for GM1. 1H-NMR also identified significantly elevated urinary excretions of bile acids, branched-chain amino acids (BCAAs), as well as their intermediates and degradation products in *NPC1* [117]. In another study, elevated lipoprotein triacylglycerol resonances were noted in both *NPC1* patients and heterozygous carriers [40], highlighting these as valuable biomarkers for disease monitoring. High-Resolution Magic Angle Spinning (HR-MAS) NMR is typically used for tissue samples, such as intact biopsies and brain sections [118], whereas in-cell NMR is a powerful approach for tracking metabolites, protein structures, and dynamics in real time [119]. Although not widely adopted, both these techniques have the potential to reveal tissue-level metabolic pathology and the molecular mechanisms behind the LSDs in their native environments.

Despite its simplicity in sample preparation and the ease of quantitation, a limiting factor that prevents wider adoption of NMR for untargeted metabolomic studies is the lack of analytical sensitivity for lower-abundant analytes. LC-HRMS approaches have an advantage in this aspect but are prone to more variability in quantitation due to ion suppression effects. In essence, these two approaches could be seen as complementary, and there is an innate value in combining them for untargeted metabolomic studies, such as those for LSDs, to enhance metabolome coverage [120,121].

### 8.4. Machine Learning and Artificial Intelligence in Untargeted Analysis

Modern high-resolution NMR spectrometers, HRMS, and faster chromatographic systems now generate data with unprecedented sensitivity, resolution, and throughput. While these technologies greatly expand metabolic coverage, they also produce vast, complex datasets that cannot be fully exploited using conventional statistical tools alone. Machine Learning (ML) algorithms have evolved from MVA statistical approaches and can be effectively applied to these datasets to detect patterns and classify disease [122,123,124]. Rather than acting as mere classification options, advanced supervised architectures—such as Random Forests (RF), Support Vector Machines (SVM), and eXtreme Gradient Boosting (XGBoost)—alongside Artificial Neural Networks (ANN) play interconnected methodological roles in high-dimensional data workflows, specifically governing feature selection, biomarker discovery, and predictive modelling. In untargeted metabolomics, tree-based ensemble methods like RF and kernel-based frameworks like SVM are extensively utilised for recursive feature elimination and metric-driven screening. This process allows researchers to filter through thousands of redundant spectral features, ranking them by predictive importance to isolate a clean, robust subset of candidate biomarkers [123,124]. For instance, a recent study by Ge et al. successfully combined untargeted serum metabolomics with an integrated ML workflow, utilising RF and SVM for robust feature selection alongside XGBoost modelling to identify high-confidence metabolic signatures capable of defining disease extent and stratification [125]. Conversely, architectures like ANNs excel in downstream predictive modelling, leveraging multi-layer perceptron frameworks to capture intricate, non-linear biological relationships and integrate multidimensional or multimodal data [123,124]. As demonstrated by Temur et al., optimised ANN frameworks can seamlessly consolidate highly heterogeneous physiological and clinical parameters into highly accurate prognostic and predictive algorithms [126]. Within the research domain of Lysosomal Storage Disorders (LSDs), while classical MVA and standard ML classifiers have already been employed for the untargeted assessment of conditions such as MPSs [49], GM1 and GM2 Gangliosidosis [32], and NPC [40], the systematic implementation of these advanced data-driven modelling workflows offers massive translational potential. By transitioning from single-metabolite metrics to robust multi-marker panels, these architectures can decode the complex, multi-systemic metabolic networks characteristic of LSD phenotypes, thereby advancing early diagnostic workflows, patient subphenotyping, and longitudinal treatment monitoring [123,124]. Following this paradigm, Deep Learning is an ML tool based on neural networks (NN) that has risen in prominence over the last decade, as it enables multiple processing layers to learn representations of data at multiple levels of abstraction [127]. It has enabled researchers to apply an integrated solution to the matching uncertainty issue and feature/metabolite selection in metabolic network analysis and predictive modelling for untargeted LC-HRMS metabolomic studies [128], while also assisting with metabolite annotation to enable generation of metabolite structures from MS2 spectra, providing de novo metabolite annotation of previously uncharacterised analytes [129]. Making use of such models could reveal new and unidentified biomarkers for understanding the impact of LSDs on cellular biochemistry and their subsequent treatment.

A challenge associated with untargeted metabolomic studies of LSDs is the rarity of the disorders, and subsequent sample availability from patients. Data augmentation (DA) is a technique that artificially increases the size and diversity of datasets, helping improve the performance of ML models in the absence of larger sample sets. Generative Adversarial Networks (GAN) are a type of DA that can learn the distribution of original data in order to generate new synthetic information to improve prediction performance [130] assisting with imbalanced datasets where sample availability is more limited. GAN was applied to 1H-NMR datasets for increasing prediction performance of *NPC1* disease activity in patients [131], providing a foundation to apply this approach in other LSDs.

## 9. Conclusions and Future Perspectives

Current approaches to NBS for LSDs and IEM generally rely on targeted screening of key metabolites and substrates, providing only a small snapshot of the patient’s status. The future of these screening programmes lies in the untargeted metabolomic approaches that widen the scope of detecting perturbations in the cellular biology of LSDs. Therefore, analytical approaches that enable the simultaneous measurement of targeted metabolites and acquisition of untargeted metabolic information in a single analysis are ideally suited to both validate current knowledge and uncover novel insights to advance NBS. Metabolomics has already had a tangible impact on the field of LSDs; in fact, it has contributed to the identification and validation of disease-specific biomarkers, several of which have been incorporated into diagnostic workflows and patient monitoring, such as lysosphingolipids in Gaucher disease and Fabry disease. These advances demonstrate that the metabolomics has evolved from a purely exploratory research tool to an approach with increasing translational and clinical relevance. In this context, untargeted metabolomics has gained increasing attention as a promising approach for advancing precision and personalised medicine, owing to its ability to comprehensively characterise metabolic alterations associated with disease susceptibility, onset, progression, and response to treatment. Its high sensitivity to physiological and pathological changes also supports its application in screening programmes for early diagnosis and patient stratification. Moreover, the integration of metabolomic data with genomic and other multi-omics datasets provides valuable insights into complex biological mechanisms, facilitating the discovery of robust biomarkers and novel therapeutic targets. Untargeted metabolomics further offers significant advantages for longitudinal patient monitoring, enabling the evaluation of metabolic trajectories before and after therapeutic interventions and supporting treatment follow-up.

Despite these considerable strengths, several challenges continue to limit the clinical translation and reproducibility of untargeted metabolomics studies. A major source of variability arises from the substantial heterogeneity of biological matrices employed across studies, including biofluids, tissues, cellular systems, and animal models, which complicates cross-study comparisons and meta-analyses. In addition, the lack of harmonised protocols for sample preparation, data acquisition, processing, and statistical analysis across laboratories remains a critical issue. Finally, metabolite annotation and identification are still hampered by the limited coverage, accessibility, and standardisation of available databases, representing a significant bottleneck for data interpretation and biological validation. Addressing these challenges will be essential to fully exploit the potential of untargeted metabolomics in both research and clinical settings. The increasing implementation of artificial intelligence and machine learning tools is expected to enhance the adoption of untargeted metabolomics workflows by improving data processing efficiency, predictive modelling, and disease classification. Furthermore, the integration of metabolomics with other omics disciplines through AI-driven approaches may help overcome the limitations of individual methodologies, enabling a more comprehensive understanding of disease biology and supporting the development of more accurate diagnostic and prognostic tools.

In conclusion, as already described throughout the review untargeted metabolomics provide huge amounts of data, for this reason successful implementation of untargeted metabolomics in routine clinical practice will also require standardised analytical workflows, robust bioinformatic pipelines, and integration with Laboratory Information Management Systems (LIMS) to ensure efficient data management, traceability, and reporting.

## Figures and Tables

**Table 1:** Sphingolipidoses: summary of untargeted metabolomic studies and associated metabolic alterations. ↑ indicates increased metabolite levels; ↓ indicates decreased metabolite levels.

Disease	Key Alterations	Sample Type	Analytical Technique	Current Diagnostic Biomarkers	Novel Biomarker Candidates
Krabbe Disease	*GALC* deficiency; PSY accumulation; profound sphingolipid and myelin lipid dysregulation; decreased HexCer, sulfatides, PC and PE-O; altered glucose and mitochondrial metabolism; oxidative stress and neuroinflammation	Nervous system tissues (brain, cerebellum, spinal cord, sciatic nerve) from murine KD models	Untargeted LC–MS/MS lipidomics, LC–MS and GC–MS metabolomics	GALC enzyme activity (first-tier screening) [9]; PSY validated diagnostic and confirmatory biomarker (DBS-based) [10]	Lipid dysregulation (HexCer, SHexCer, Cer, PC, PE-O, sphingolipids) [8]; lipid network disruption linked to PSY accumulation [8]; energy metabolism dysfunction (acyl-carnitines, BCAA) [11]; neuronal damage marker (NAA) [11]; glycerophospholipid metabolism alterations (GPC, CDP-choline, G3P) [11]; neuroinflammation/oxidative stress markers (12-HETE, corticosterone, α-tocopherol) [11]
Gaucher Disease	*GBA* deficiency; glucosylceramide and Lyso-Gb1 accumulation; neuroinflammation with increased miR-155 expression; impaired osteoblast differentiation and dysregulated Wnt signalling	Zebrafish *GBA1* loss-of-function models; DBS	TALEN-mediated disease modelling; enzymatic assay; targeted sphingolipid analysis	GBA enzyme activity (first-tier screening); Lyso-Gb1 validated second-tier diagnostic biomarker (DBS/plasma) [16,17,18]	Sphingolipid-related metabolites (Lyso-Gb1 analogues, sphingosylphosphorylcholine, N-palmitoyl-O-phosphocholineserine) [19]; inflammation-associated circulating molecular markers (miRNA-based signatures, including miR-155) [15] Skeletal-related molecular signatures linked to lysosomal dysfunction (Wnt pathway-associated readouts in preclinical models) [15]
Fabry Disease	*GLA* deficiency; Gb3 and Lyso-Gb3 accumulation; altered glycosphingolipid metabolism; increased Lyso-Gb3 analogues, Ga2 isoforms, and ceramide dihexosides; oxidative stress, inflammation, and glycerophospholipid remodelling	Urine; plasma	LC–MS/MS glycosphingolipidomics; LC–TOF-MS/MS metabolomics; network-based targeted metabolomics	GLA enzyme activity (first-tier diagnostic test) [20,21,22]; Lyso-Gb3 and related analogues (diagnostic and therapeutic monitoring biomarkers) [22,26]	Urinary glycosphingolipid signatures (Lyso-Gb3 analogues, Ga2 isoforms, ceramide dihexosides) [22,23]; plasma metabolomic and lipid remodelling signatures (including methylated Gb3 derivatives and oxidative stress–related metabolites) [23]; circulating inflammatory and cardiac biomarkers (TNF, IL-6, MMPs, NT-proBNP) [23,26]
Metachromatic Leukodystrophy	ASA deficiency leading to accumulation of sulfatides (especially 3-O-sulfogalactosylceramides); progressive lysosomal dysfunction, membrane damage, oligodendrocyte death, demyelination, and neuroinflammation; extensive lipid remodelling including increased sulfatides (notably short-chain SHexCer ≤ C18), HexCer, and altered ceramide balance (↓ Cer_NS, ↓ Cer_NDS, Cer_AS); decreased fatty acids, FAHFAs, diacylglycerols (DG), and triacylglycerols (TG); increased LPCs and acylcarnitines; reduced phospholipids (PE, PS, PI, PC) with alterations in PG and BMP; metabolic signatures consistent with mitochondrial dysfunction, impaired energy metabolism and inflammatory activation	Brain-derived extracellular vesicles (EVs) from *ARSA*^−^/^−^ mouse model; urine, plasma, and CSF from human patients; DBS for newborn screening	Untargeted LC–MS/MS lipidomics (EVs and tissues); LC–MS/MS sulfatide quantification (DBS screening); ^1^H-NMR metabolomics (urine); magnetic resonance spectroscopy (MRS) with metabolite profiling; enzymatic ASA activity assays and genetic testing	ASA enzyme activity (diagnostic screening) [27,28]; C16:0 sulfatide DBS as a newborn screening biomarker [27,30,31]; urinary sulfatides (3-O-sulfogalactosylceramides) as biochemical biomarkers [27,28]	Sphingolipid and lipid remodelling markers (SHexCer, ceramides, HexCer, LPC, FAHFAs, acylcarnitines) [29]; neurodegeneration-associated markers (NAA, lactate, glutamate alterations) [27,28]; systemic and microbiota-related metabolic signatures (TMA, 2-furoylglycine, L-citramalic acid) [28]
GM1- Gangliosidoses	Deficiency of β-galactosidase leading to accumulation of GM1 ganglioside and related substrates; systemic metabolic reprogramming affecting amino acid, lipid, and energy metabolism; increased amino acids (BCAA: valine, leucine, isoleucine; aromatic amino acids: tyrosine, phenylalanine; glutamine, histidine); elevated lactate, formate, and urea indicating shift toward anaerobic metabolism; altered creatine/phosphocreatine/creatinine metabolism; reduced triglycerides; mitochondrial dysfunction, oxidative stress, impaired β-oxidation and TCA cycle	Plasma, cerebrospinal fluid (CSF); urine (human patients, including GM1 type 2 cohort)	^1^H NMR-based metabolomics with multivariate statistical analysis and metabolite set enrichment/pathway analysis	GLB1 enzyme activity [32]	Plasma amino acid and energy metabolism signatures (BCAAs, lactate, creatine-related metabolites) [32]; lipid metabolism and mitochondrial dysfunction signatures [32]
GM2- Gangliosidoses	Defects in β-hexosaminidase system leading to accumulation of GM2 and related gangliosides in the CNS; progressive neurodegeneration with neuronal loss, glial activation, and neuroinflammation; elevated CSF inflammatory mediators (e.g., MCP-1, MIP-1α/β, ENA-78, TNFR2, IGFBP-2); altered lipid metabolism including accumulation of GM2/GA2 gangliosides, BMPs, and sphingosine; decreased HexCer and polyunsaturated fatty acids; widespread metabolic reprogramming with reduced NAA, glutamate, aspartate, and GABA; increased myo-inositol, oxidised glutathione, and aminoadipic acid; impaired mitochondrial function, oxidative stress, and endolysosomal dysfunction	CSF and serum from patients; brain, spinal cord, liver, and hippocampus from SD models and human tissue samples	Untargeted LC–MS/MS metabolomics (brain, liver, hippocampus); lipidomics of sphingolipids and BMPs; immunoassays for inflammatory cytokines in CSF/serum; metabolite profiling of neurotransmitters and oxidative stress markers	β-Hexosaminidase A/B enzymatic activity [33,34,35]	Neuroinflammatory signatures (MCP-1, MIP-1α, ENA-78, TNFR2, osteopontin, IGFBP-2) [33]; lipidomic signatures of lysosomal dysfunction (BMPs, sphingosine, lysophospholipids, GM2/GA2) [34,35]; neurodegeneration and metabolic dysfunction signatures (NAA, glutamate, GABA, oxidised glutathione) [34,35]
Niemann-pick Type C Disease	Impaired intracellular cholesterol trafficking leading to lysosomal accumulation of unesterified cholesterol and secondary glycosphingolipid storage; widespread endolysosomal dysfunction with accumulation of GM2/GM3 gangliosides, monohexosylceramides, sphingoid bases, and elevated BMPs (e.g., BMP 22:6/22:6); reduced myelin-associated lipids (plasmalogen PE, HexCer); increased sphingosine and lysophospholipid dysregulation (notably alkyl-LPCs such as LPC O-16:0, O-18:1, O-18:0); broader metabolic reprogramming involving amino acid, creatine, and redox pathways; altered bile acid metabolism with accumulation of oxysterol-derived bile acids; impaired mitochondrial function, oxidative stress, and synaptic dysfunction	Brain, liver, spleen, and other peripheral tissues from *NPC1/NPC2* models; plasma, CSF, urine, and DBS from patients	Untargeted and targeted LC–MS/MS lipidomics; sphingolipid and sterol profiling; oxysterol/bile acid quantification (DBS, plasma); ^1^H-NMR metabolomics (plasma, urine); CSF lipidomics; multivariate and pathway analysis approaches	Oxysterol-derived bile acids (3β,5α,6β-trihydroxycholanic acid and glycine conjugate) [39]	Lipidomic signatures of lysosomal dysfunction (BMPs, alkyl-LPCs, sphingolipids, gangliosides) [37,38]; circulating metabolomic signatures of amino acid, energy and redox metabolism [40,41,42]
Acid Sphingomyelinase Deficiency	ASMD leading to progressive sphingomyelin accumulation and multisystem lipid storage affecting spleen, liver, lungs, bone marrow, and lymph nodes, with CNS involvement in severe forms; systemic lipid dyshomeostasis associated with oxidative stress, lipid peroxidation, and membrane damage; clinical heterogeneity ranging from severe neurovisceral infantile disease (type A) to chronic visceral phenotypes (type B); metabolic signatures reflecting pulmonary involvement and disease severity; altered VOC profiles linked to oxidative lipid degradation and inflammatory processes	Exhaled breath and exhaled breath condensate from adult chronic visceral ASMD patients; tissues (spleen, liver, lung, bone marrow) in clinical context	GC–MS and LC–MS-based volatilomics (exhaled breath); multivariate statistical analysis for pattern recognition and disease stratification; correlation with imaging (HRCT) and pulmonary function (DLCO)	Acid sphingomyelinase enzyme activity [43,44,45] Lyso-SM and Lyso-SM-509 (diagnostic biomarkers) [43,44,45]	Exhaled breath volatile organic compound (VOC) signatures associated with oxidative stress, lipid peroxidation and pulmonary involvement [46]; VOC-based metabolomic signatures for disease stratification and therapeutic monitoring [46]

**Table 2:** Mucopolysaccharidoses: untargeted metabolomic studies and associated metabolic signatures.

Disease	Key Alterations	Sample Type	Analytical Technique	Current Diagnostic Biomarkers	Novel Biomarker Candidates
MPS I (Hurler, Hurler–Scheie, Scheie Syndrome)	α-L-iduronidase deficiency with impaired catabolism of Dermatan and Heparan sulfate	Urine	LC-MS/MS for the detection of Non-Reducing Trisaccharides (NREs); LC-HRMS studies for metabolic remodelling	*IDUA* enzyme activity [50,51]; NRE trisaccharides (diagnostic and monitoring biomarkers) [50,51]; urinary (GAGs) quantification [50,51]	Untargeted metabolomic signatures of amino acid, carbohydrate, lipid and oxidative stress dysregulation [49]; proteomic signatures of extracellular matrix remodelling, inflammation and fibrosis (lung involvement) [52];
MPS II (Hunter Syndrome)	Deficiency of iduronate-2-sulfatase leading to accumulation of Heparan and Dermatan sulfate	Urine	LC-MS/MS	*IDS* enzyme activity [47,48]; urinary GAGs (heparan sulfate and dermatan sulfate) quantification [47,48]	Gangliosides (GM2, GM3) and ceramides as biomarkers of sphingolipid dysregulation and neurodegeneration [53]; metabolomic signatures of histidine, tryptophan, and energy metabolism dysregulation as indicators of systemic metabolic imbalance [54]
MPS III (Sanfilippo Syndrome)	MPS III has 4 subtypes (A–D), each caused by a defect in a different enzyme involved in the catabolism of heparan sulphate	Urine	LC-MS/MS	Enzyme activity (depending on the subtype) [55]; urinary heparan sulfate quantification [55]	Metabolomic signatures of membrane lipid remodelling, arginine metabolism dysregulation, oxidative stress, and mitochondrial bioenergetic impairment as indicators of systemic metabolic dysfunction [56]; neurofilaments, sphingolipids, and inflammatory markers as candidate biomarkers of neurological disease progression [55]
MPS IV (Morquio Syndrome)	Deficiency of lysosomal enzymes leading to accumulation of Keratan sulfate (type A and B MPS IV)	Urine	LC-MS/MS	*GALNS* or *GLB1* enzyme activity (depending on the subtype) [48]; urinary keratan sulfate quantification [48]	Inflammatory markers and extracellular matrix proteins [48]
MPS VI (Maroteaux–Lamy Syndrome)	Arylsulfatase B deficiency leading to impaired Dermatan sulfate degradation	Urine	LC-MS/MS	*ARSB* enzyme activity [57]; urinary GAGs (dermatan sulfate) quantification [57]	Untargeted metabolomic signatures of dysregulation in energy, fatty acid and aromatic amino acid metabolism, and oxidative stress [57]
MPS VII (Sly Syndrome)	Deficiency of β-glucuronidase leading to accumulation of Dermatan, Heparan and Chondroitin sulfate	Urine	LC-MS/MS	β-glucuronidase (*GUSB*) enzyme activity [58]; urinary GAGs quantification (dermatan, heparan and chondroitin sulfate) [58]	No untargeted metabolomics studies available [58]

**Table 3:** Lysosomal glycogen storage disease (Pompe disease): overview of untargeted metabolomic alterations and potential biomarkers.

Disease	Key Alterations	Sample Type	Analytical Technique	Current Diagnostic Biomarkers	Novel Biomarker Candidates
Lysosomal Glycogen Storage Disease: Pompe Disease	Acid α-glucosidase deficiency with lysosomal glycogen accumulation; autophagy impairment; AMPK/mTOR dysregulation; oxidative stress; mitochondrial dysfunction; altered glycolysis and lipid β-oxidation; altered calcium homeostasis	Urine, plasma/serum, skeletal muscle biopsy, fibroblasts, cardiomyocytes	Metabolomics, proteomics, lipidomics, transcriptomics, enzymatic assay, multi-omics integration	Acid α-glucosidase (GAA) enzyme activity [59,60]; urinary glycogen-derived tetrasaccharide (Glc4) [60,61]; urinary oligosaccharides (Hex5, Hex6, Hex7) as multi-metabolite diagnostic panel [61]	Proteomic and lipidomic signatures of glycolytic and lipid metabolism dysregulation (e.g., LDHB, PKM, GPLD1, PON1) [61,62]; Metabolomic alterations in energy metabolism, including glycolytic depletion and increased Krebs cycle intermediates and fatty acids [62]; Integrated proteomic–metabolomic candidates (e.g., CALML3, NEFL) [63]

**Table 4:** Oligosaccharidoses: characterisation of metabolic dysregulation.

Disease	Key Alterations	Sample Type	Analytical Technique	Current Diagnostic Biomarkers	Novel Biomarker Candidates
α-mannosidosis	α-mannosidase deficiency; accumulation of mannose-rich oligosaccharides; progressive neurodegeneration, intellectual disability, skeletal abnormalities, hearing loss	Urine; plasma	MS-based oligosaccharide profiling; targeted metabolomics	α-mannosidase enzyme activity [67]; urinary mannose-containing oligosaccharides accumulation [67]	No untargeted metabolomics studies available
β-mannosidosis	β-mannosidase deficiency; accumulation of mannose-containing oligosaccharides	Urine	Mass spectrometry (oligosacchariduria profiling)	β-mannosidase enzyme activity [68]; urinary oligosaccharideuria and MS-based oligosaccharide analysis [68]	No untargeted metabolomics studies available
Fucosidosis	α-L-fucosidase deficiency; accumulation of fucosylated glycoconjugates; neurodegeneration, angiokeratomas, hepatosplenomegaly	Urine	MS-based glycan/oligosaccharide analysis	α-L-fucosidase enzyme activity [69]; urinary fucosylated substrates accumulation [69]	No untargeted metabolomics studies available
Sialidosis	neuraminidase deficiency; accumulation of sialylated oligosaccharides; type I (mild) to type II (severe) phenotypes	Urine; fibroblasts	Mass spectrometry; enzymatic assays	Neuraminidase enzyme activity [70]; urinary sialylated oligosaccharides accumulation [70]	No untargeted metabolomics studies available
Galactosialidosis	combined neuraminidase and β-galactosidase deficiency; accumulation of mixed glycoproteins and oligosaccharides	Urine	MS-based metabolomics; glycan profiling	Combined neuraminidase and β-galactosidase deficiency [71]; urinary glycoproteins and oligosaccharides accumulation [71]	No untargeted metabolomics studies available
Aspartylglucosaminury	aspartylglucosaminidase deficiency; accumulation of glycoasparagine and related glycopeptides	Urine	Mass spectrometry; targeted metabolomics	Glycosylasparaginase (AGA) enzyme activity [72]; urinary glycoasparagine and related glycopeptides accumulation [72]	No untargeted metabolomics studies available

**Table 5:** Lysosomal proteinoses: current evidence from untargeted metabolomic studies and associated metabolic alterations.

Disease	Key Alterations	Sample Type	Analytical Technique	Current Diagnostic Biomarkers	Novel Biomarker Candidates
Cystinosis	Reduced alanine, aspartate, glutamate, arginine, methionine, and glutathione metabolites; altered sulfur and redox metabolism; increased fumarate, malate, and citrate turnover indicating mitochondrial and TCA cycle dysfunction; dysregulated apoptosis- and ribosome-related proteins	*CTNS*-silenced HK-2 cell model	Integrated multi-omics approach combining untargeted metabolomics, fluxomics, and proteomics	Leukocyte cystine concentration [73]; ophthalmologic detection of corneal cystine crystals [73]	Integrated metabolomic, fluxomic and proteomic signatures of altered amino acid (alanine, aspartate, glutamate, arginine, methionine), sulfur, glutathione and energy metabolism [77]; alterations in mitochondrial and Krebs cycle metabolites (fumarate, malate, citrate) [77]; proteomic signatures of dysregulated apoptosis, ribosome biogenesis and protein synthesis [77]
Neuronal Ceroid Lipofuscinosis 2	Reduced acetylated metabolites acetylaspartylglutamate (NAAG), N-acetylneuraminic acid and its dimer, N-acetylalanine, N-acetylserine, N-acetylthreonine, glycerol-3-phosphoinositol, and sulfoacetic acid; altered neuronal and energy metabolism; strong correlation with neurodegeneration severity	Cerebrospinal fluid (CSF) from *CLN2* patients	Untargeted LC–MS-based metabolomics	Tripeptidyl peptidase 1 (TPP1) enzyme activity [80]	Untargeted CSF metabolomic signature with decreased N-acetylated metabolites (including N-acetylaspartylglutamate, N-acetylneuraminic acid, N-acetylalanine, N-acetylserine, N-acetylthreonine) [80]; reduced glycerol-3-phosphoinositol and sulfoacetic acid levels associated with disease severity and progression [80]
Neuronal Ceroid Lipofuscinosis 6	Reduced phosphatidylglycerols (PG 40:6, PG 40:7) and glucosylceramides (C16 GlcCer, C24 GlcCer, C24:1 GlcCer); dysregulated sphingolipid and glycerophospholipid metabolism; altered calcium signalling and neuronal homeostasis	Human *CLN6*-derived neuronal cells generated from patient fibroblasts (ciNPCs)	Untargeted LC–MS-based metabolomics	Clinical diagnosis based on neurodevelopmental features (ataxia, seizures, progressive cognitive decline) [83]	Untargeted LC–MS metabolomic signature in patient-derived neuronal cells showing dysregulation of lipid metabolism, with alterations in sphingolipid and glycerophospholipid pathways and downregulation of key lipid species (phosphatidylglycerols and glucosylceramides) [83]
Danon Disease	Altered glycolysis and TCA cycle metabolism; mitochondrial dysfunction; impaired oxidative phosphorylation; dysregulated acylcarnitine and NAD^+^ pathways; inflammatory and fibrotic remodelling	Cardiac tissue, hiPSC-cardiomyocytes, and fibroblasts	Integrated transcriptomics and untargeted metabolomics	Muscle biopsy showing vacuolated myocytes with autophagic material accumulation [86]; cardiac imaging and clinical phenotype assessment (cardiomyopathy, myopathy, arrhythmias) [86]	Integrated transcriptomic and untargeted metabolomic signatures showing metabolic reprogramming, glycolytic shift, mitochondrial dysfunction, altered NAD^+^ metabolism, and myocardial remodelling [87]
Lysosomal Cathepsin Deficiencies	Impaired lysosomal proteolysis and autophagy with accumulation of undegraded proteins and disrupted proteostasis; secondary neurodegenerative and lysosomal dysfunction effects	Cellular/experimental models and tissues	Proteomic and enzymatic assays; autophagy flux studies	Not reported	No untargeted metabolomics studies available

**Table 6:** Mucolipidoses: insights into metabolic perturbations revealed by untargeted metabolomics.

Disease	Key Alterations	Sample Type	Analytical Technique	Current Diagnostic Biomarkers	Novel Biomarker Candidates
Mucolipidosis type II	Severe lysosomal trafficking defect leading to multisystem accumulation of undegraded substrates; prenatal-onset disease with dysostosis multiplex, growth restriction, coarse facial features, organomegaly, joint contractures, and cardiopulmonary complications; profound neurodevelopmental impairment and early mortality	Prenatal (including hydrops fetalis) and postnatal clinical tissues; imaging and systemic evaluation	Clinical and radiological assessment; enzymatic assays (lysosomal hydrolases); genetic testing	UDP-GlcNAc-1-phosphotransferase activity assay [92]; increased lysosomal enzyme activities in plasma, DBS, and fibroblast/amniocyte culture media [92]; mannose-6-phosphate (M6P)-containing protein assays [92]	No untargeted metabolomics studies available
Mucolipidosis type III	Defect in lysosomal trafficking with intracellular accumulation of lysosomal enzymes; reduced mannose-6-phosphate (M6P) tagging → misrouting and secretion of lysosomal enzymes instead of lysosomal targeting; progressive multisystem dysfunction	Plasma, fibroblasts, leukocytes, urine	Lysosomal enzyme activity assays (fibroblasts/leukocytes), measurement of extracellular lysosomal enzymes in plasma, molecular genetic testing (*GNPTAB*/*GNPTG*), cellular studies in fibroblasts	UDP-GlcNAc-1-phosphotransferase activity assay [92]; increased lysosomal enzyme activities in plasma, DBS, and fibroblast/amniocyte culture media [92]; mannose-6-phosphate (M6P)-containing protein assays [92]	No untargeted metabolomics studies available

## Data Availability

No new data were created or analysed in this study.
